# A National Case-Control Study Identifies Human Socio-Economic Status and Activities as Risk Factors for Tick-Borne Encephalitis in Poland

**DOI:** 10.1371/journal.pone.0045511

**Published:** 2012-09-19

**Authors:** Pawel Stefanoff, Magdalena Rosinska, Steven Samuels, Dennis J. White, Dale L. Morse, Sarah E. Randolph

**Affiliations:** 1 Department of Epidemiology, National Institute of Public Health – National Institute of Hygiene, Warsaw, Poland; 2 State University of New York at Albany, School of Public Health, Rensselaer, New York, United States of America; 3 New York State Department of Health, Albany, New York, United States of America; 4 National Center for Emerging and Zoonotic Infectious Diseases, Centers for Disease Control and Prevention, Atlanta, Georgia, United States of America; 5 Department of Zoology, University of Oxford, Oxford, United Kingdom; University of Minnesota, United States of America

## Abstract

**Background:**

Tick-borne encephalitis (TBE) is endemic to Europe and medically highly significant. This study, focused on Poland, investigated individual risk factors for TBE symptomatic infection.

**Methods and Findings:**

In a nation-wide population-based case-control study, of the 351 TBE cases reported to local health departments in Poland in 2009, 178 were included in the analysis. For controls, of 2704 subjects (matched to cases by age, sex, district of residence) selected at random from the national population register, two were interviewed for each case and a total of 327 were suitable for the analysis. Questionnaires yielded information on potential exposure to ticks during the six weeks (maximum incubation period) preceding disease onset in each case. Independent associations between disease and socio-economic factors and occupational or recreational exposure were assessed by conditional logistic regression, stratified according to residence in known endemic and non-endemic areas. Adjusted population attributable fractions (PAF) were computed for significant variables. In endemic areas, highest TBE risk was associated with spending ≥10 hours/week in mixed forests and harvesting forest foods (adjusted odds ratio 19.19 [95% CI: 1.72–214.32]; PAF 0.127 [0.064–0.193]), being unemployed (11.51 [2.84–46.59]; 0.109 [0.046–0.174]), or employed as a forester (8.96 [1.58–50.77]; 0.053 [0.011–0.100]) or non-specialized worker (5.39 [2.21–13.16]; 0.202 [0.090–0.282]). Other activities (swimming, camping and travel to non-endemic regions) reduced risk. Outside TBE endemic areas, risk was greater for those who spent ≥10 hours/week on recreation in mixed forests (7.18 [1.90–27.08]; 0.191 [0.065–0.304]) and visited known TBE endemic areas (4.65 [0.59–36.50]; 0.058 [−0.007–0.144]), while travel to other non-endemic areas reduced risk.

**Conclusions:**

These socio-economic factors and associated human activities identified as risk factors for symptomatic TBE in Poland are consistent with results from previous correlational studies across eastern Europe, and allow public health interventions to be targeted at particularly vulnerable sections of the population.

## Introduction

Tick-borne encephalitis (TBE) is the most significant vector-borne viral infection in Europe, with clinical symptoms that commonly involve the central nervous system, leading to a high percentage of neurological sequelae (c.25%), psychiatric problems (c.45%), and fatality in c.1% of the 3–4000 annual cases [1]. Its focal distribution across much of Europe, from eastern France to the Baltic countries (and through much of Russia) and Sweden to the Balkans [2], [3], is related to persistent natural enzootic cycles vectored by ticks (principally *Ixodes ricinus* and also *I. persulcatus* in the east) amongst transmission-competent rodents (principally *Apodemus* species [4]), for which specific environmental conditions are required. As *Ixodes* ticks are very sensitive to desiccation, humidity must remain high through the summer for good tick survival and questing activity [5], [6]. Furthermore, a relatively rapid rate of increase in spring temperatures is necessary to allow maximal synchrony in the activity of larval and nymphal ticks and thereby a high degree of co-feeding by these stages on rodents, essential for TBEV transmission [7], [8], [9]. In addition to rodents, large hosts such as deer are essential to support tick populations, feeding significant numbers of both immature life stages as well as adults [6], although locally very high deer densities appear to reduce TBE prevalence in rodents, perhaps because deer divert ticks from feeding on rodents [10]. These abiotic and biotic constraints make forests the principal habitat for infected ticks, which has important consequences for risk factors.

Human infections arise principally through tick bites to which people are exposed as they enter the forests for occupation and recreation. Geographically variable patterns of increase in TBE incidence have occurred in most parts of Europe: gradual but significant increases, including the emergence of new foci, have occurred in western and northern countries over the past two-three decades [11], [12], [13], [14], in contrast to abrupt upsurges in erstwhile communist countries in the early 1990s [15]. The latter was particularly marked in Poland, where annual case numbers increased by an order of magnitude from 1992 to 1993 and have been maintained at this high level ever since (mean +/− st. dev. annual cases 1975–1992, 21+/−14; 1993–2010, 229+/−69) (see Fig. 1 in [15]).

**Figure 1:**
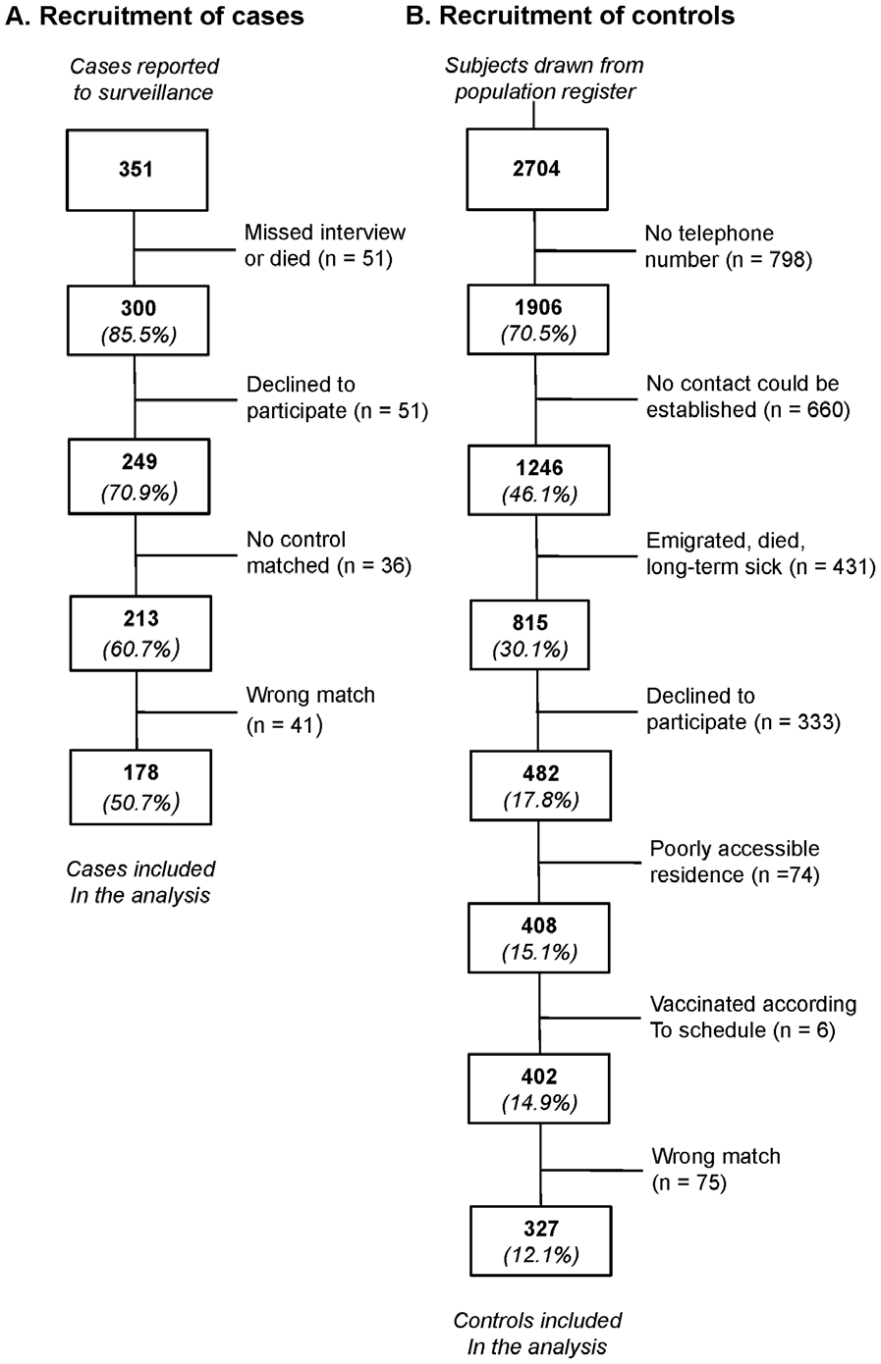
Summary of recruitment process. Case-control study of tick-borne encephalitis risk factors, Poland, January–December 2009.

Recent studies to assess the factors associated with the occurrence and upsurge of TBE have mostly been of an ecologic design, identifying correlates in time and space within a biologically and epidemiologically plausible framework. Some factors act directly on the enzootic cycle, but those that act on the degree of human exposure to infected ticks can cause more abrupt, spatially differential changes [16]. In the Czech Republic, any effect of socio-economic factors on exposure has been denied [17], [18], despite the largest proportional increase in incidence post-1992 occurring in people aged over 65 years (see Fig. 5 in [17]). Instead, climate change has been emphasized as the sole causal factor [18], although marked heterogeneities at regional and even very fine geographical scales make this explanation untenable [19]. Although it is likely that increased incidence at higher altitudes in Austria, Slovakia and the Czech Republic [20], [21], [22] reflects warmer temperatures under limiting conditions along the distributional boundaries, the case numbers at these mountainous sites cannot account for either the full amount or the geographical pattern of the increases in incidence across central Europe. Instead, changes in specific climatic factors [23], in landscape resources and their utilization [9], [24], and, most markedly, in socio-economic conditions that accompanied the transition to free-market economies [15], [25], have all been identified as part of a network of independent but synergistic factors significantly correlated with TBE incidence. Each factor will operate with differential force and on different time-scales depending on the cultural, societal and political contexts characteristic of each country. Gradual increases in TBE incidence in western countries that have not experienced extreme political changes do not, of course, deny the role of slower socio-economic evolution in those countries (e.g. more outdoor recreation by retired people [26]) or abrupt socio-economic transitions in ex-communist countries, despite assertions to the contrary [27]. Furthermore, short-term changes in the weather in one case (2006) and the recent economic crisis in another (2009) have been shown to explain annual spikes in incidence via their effects on human behaviour [28], [29], [30]. Changes in public health services, however, have been discounted as a sufficient explanation [31].

The aim of the present study is to test the credibility of the emergent explanations based on correlations by applying a more rigorous analytical epidemiological study at the individual level to assess associations between specific risk factors and disease. This was achieved by conducting a nationwide case-control study for Poland, the first such study for TBE, to compare the socio-economic status, residence characteristics, travel history and outdoor exposure to tick bites between TBE cases diagnosed during 2009 and randomly selected members of the population. The additional aim was to differentiate risk arising from exposure incurred through occupation or recreation, including travel-related risk. Knowledge of individual risk factors is particularly important for TBE because, in the absence of any specific antiviral treatment [1], prophylactic interventions are the only means for limiting human transmission. These include landscape management to control tick abundance, education about personal protective measures to reduce exposure to ticks, and vaccination using one of the two highly effective vaccines (produced by Baxter and Novartis) [32]. Public health interventions can be much better targeted if high quality information exists on the risk factors likely to make some sections of the population particularly vulnerable.

## Methods

### Ethics Statement

The study protocol received written approved from the Ethical Committee of National Institute of Public Health – National Institute of Hygiene. Written consent was obtained from each adult subject and written consent of the legal guardian was obtained for each minor (person under 18 years of age). All consent forms are stored at the Department of Epidemiology of the National Institute of Public Health in Warsaw.

### Study Design

The population-based, national case-control study to assess TBE risk factors covered ten of the 16 Polish provinces. The decision to set up the study in any particular province, and to recruit a network of interviewers with regional coordinators, was based on the expected occurrence of TBE cases (at least five TBE cases reported annually during the previous five years or their prior inclusion in a parallel screening study, in which all patients with aseptic CNS infection were tested for TBE). The study was performed by a team of national coordinators, with two regional coordinators in each province, and 90 trained interviewers. Face-to-face interviews were performed with all eligible subjects.

### Case Subjects

Attempts were made to recruit each diagnosed TBE case reported to the surveillance system. The Polish surveillance system has national coverage and is based on mandatory passive reporting of cases that develop symptoms of meningo-encephalitis. The system has fair sensitivity overall (48%), but diagnosis of TBE may be different in known endemic regions and the remaining parts of the country [33]. A standardized case definition is used to classify each reported case [34], as follows: a possible case is one that presents with symptoms of meningo-encephalitis, and had visited an endemic area during April-November; a probable case is one that presents with symptoms of meningo-encephalitis and either the presence of an epidemiological link (consumption of raw dairy products) or detection of IgM in serum by enzyme-linked immunosorbent assay (ELISA); a confirmed case is one that presents with symptoms of meningo-encephalitis and laboratory confirmation (IgM and IgG detection in serum, or detection of antibodies in CSF, or confirmation by neutralization test independently from other test results). All eligible cases in this study met the surveillance definition of a probable or confirmed TBE case, were not vaccinated against TBE according to the recommended schedule in the previous 5 years, had disease onset between January 1, 2009 and December 31, 2009, and gave informed consent to participate in the study. Each case was interviewed either in the hospital, or at home after discharge using a 4-page questionnaire on exposure.

### Control Subjects

Two control subjects were selected for each case, matched by sex, age (+/−5 years), and district of residence. To allow prospective selection of controls, a stratified random sample of 500 inhabitants from each studied district was obtained from the national population register, prior to the recruitment of cases. The district samples were weighted using the age-and-gender distribution typical for TBE cases reported to surveillance during the previous 20 years. After a case was notified, seven subjects meeting the matching criteria were selected at random, and contact information from the population register was updated. The regional coordinators appointed interviewers, taking into account their availability and logistic constraints related to the subject’s residence. For each case, the aim was to interview two of the selected controls that met the eligibility criteria. If the subject declined to participate in the study another control subject was selected from the list.

### Interviews

One questionnaire was used in the interview with adults and adolescents and a separate questionnaire was used in the interview with children of 12 years and younger in the presence of their parents or legal guardians. Interviews of adult subjects comprised approximately 30 questions and took about 30 minutes. Interviews of children were shorter (approximately 20 questions) but lasted longer because both the child and its parent or legal guardian were questioned. Interviewers had received 5-hour training sessions from the study coordinator, including an introduction to the study procedures and interview techniques. In addition to basic demographic data, information was sought specifically on exposure to ticks (i.e. time spent within various habitats) related to occupational and recreational outdoor activities. Interviewers were equipped with regional maps to mark geographic locations of exposure. Both cases and matched controls were asked about exposure that had occurred during a six-week period (maximum disease incubation time) preceding the onset of disease in the respective case subject. This ‘matching by exposure period’ created the potential for differential recall bias, as the recall period for control subjects was delayed by the time needed for their recruitment and the arrangement of their interview. To address this issue, interviewers used a calendar marked with important national and local events, anniversaries, festivals, and asked about important dates from the respondents’ lives to help them recall diverse activities over the relevant six-week period.

### Data Management

For the analysis, pairs were excluded if the recall period for the control covered less than 50% of the actual six-week exposure period for the case, or if controls were not adequately matched to the cases on other variables (i.e. gender, age, region of residence).

Information on occupation was collected using free text, which was then re-coded according to ISCO-08 major groups (Table 1), except for forestry workers who were retained as a separate group as they are at higher risk of exposure to ticks [35]. Children aged <16 years, the unemployed, the retired and students were originally separate groups. Due to limited sample sizes, some occupational groups were later further amalgamated if odds ratios did not differ significantly in preliminary univariate analyses.

**Table 1:** Description of variables used in the analysis, national case-control study of TBE risk factors, Poland, January–December 2009.

Variable	Categories in questionnaire	Categories used in analysis	Comments
Urbanization level	village; town <100000 inhabitants;city >100000	Original categories	
Education	child <16, primary; vocational; high school; university	0. not graduated from high school;1. high school or higher	
Income per household member	<500 PLN; 500–999 PLN;1000–1500 PLN; >1500 PLN	Original categories	Recalculated into US dollars based on average currency exchange rate in 2009
Occupation	Free text	ISCO-08 occupational groups: 1. Managers;2. Professionals; 3. Technicians and associateprofessionals; 4. Clerical support workers;5. Service and sales workers; 6. Skilled agricultural,forestry and fishery workers; 7. Craft and relatedtrade workers; 8. Plant and machine operators,and assemblers; 9. Elementaryoccupations; 10. Armed forces occupations	Elementary occupations include: cleaners and helpers; labourers in mining, construction, manufacturing and transport; food preparation assistants, street and related sales and service workers; refuse workers and other elementary workers
Immunisation status	Dates and brand names of vaccines	0 – not vaccinated; 1 – inadequately vaccinated;2 – vaccinated (3 primary doses within 3 yearsor booster dose within 5 years)	Categories based on vaccines Summaries of Product Characteristics (SPC) recommended schemes
Forest proximity (from place of residence)	<50 m; 50–100 m; 100–500 m;500–1000 m; >1 km	0. ≤500 m; 1. >500 m	Decision on final category based on variable distribution and BIC/AIC criteria
Living on a farm	Yes; No	0. No; 1. Yes	
Goats on the farm	Yes; No	0. No; 1. Yes	
Sheep on the farm	Yes; No	0. No; 1. Yes	
Cows on the farm	Yes; No	0. No; 1. Yes	
Living in a house with a yard or garden	Yes; No	0. No; 1. Yes	
Yard/garden securedfrom wild animals	Yes; No	0. No; 1. Yes	
Wild animals ever seenin yard/garden	Yes; No	0. No; 1. Yes	
In-country travel to endemic region	In country travel (Yes; No), Travel Destination (Text), District statistical number (TERYT), Latitude andLongitude from map	0. No; 1. Yes	Data for analysis combined from information on up to two travel events during exposure period. Endemic status of the travel destination (administrative district) ascertained based on 2004–2008 surveillance
In-country travel tonon-endemic region	In country travel (Yes; No), Travel Destination (Text), District statistical number (TERYT), Latitude andLongitude from map	0. No; 1. Yes	Data for analysis combined from information on up to two travel events during exposure period. Endemic status of the travel destination (administrative district) ascertained based on 2004–2008 surveillance data
Time spent travelling during exposure period	In country travel (Yes; No); Dates oftravel (date of start/date of return)	0. no travel; <5 days; 5–15 days; > = 15 days	Cumulative time from up to two travels reported
Travel distance	Town of residence: In country travel(Yes/No); Travel Destination (Text),District statistical number(TERYT), Latitude andLongitude from map	0. near residence <50 km or no travel;1. ≥50 km travel to endemic region;≥50 km travel to non-endemic region	The residence and travel destination were point mapped. The distance from residence to the travel destination was computed using ArcView software, using the function Table to Point and Geodesy Calculator. In case of two travels, the longer distance was selected
Travel abroad	Travel abroad (Yes; No), Country of Destination (Text), Dates of travel	0. No; 1. Yes	
Recreation: hunting	Yes; No	0. No; 1. Yes	
Recreation: camping	Yes; No	0. No; 1. Yes	
Recreation: fishing	Yes; No	0. No; 1. Yes	
Recreation: swimming outdoors	Yes; No	0. No; 1. Yes	
Recreation: sailing	Yes; No	0. No; 1. Yes	
Recreation: hiking	Yes; No	0. No; 1. Yes	
Recreation: cycling	Yes; No	0. No; 1. Yes	
Recreation: collecting mushrooms, berries or other forest foods	Yes; No	0. No; 1. Yes	
Recreation: gardening	Yes; No	0. No; 1. Yes	
Time spent outdoors	Hours per week in different habitats - deciduous forest, coniferous forest, mixed forest, forest edge, meadows/high grass, town parks, city streets, cottage gardens, field/farms; Original categories: 0 h;1–10 h; 11–20 h; 20–30 h; 30–40 h; >40 h	0. <10 hours; 1. ≥10 hours	Scale used in many questionnaire items: for outdoor time spent in different habitats separately in relation to work and recreation. Different aggregations were used separating occupational from recreational time, as well as combining time spent outdoors
Consumption of unpasteurized cow milk or cheese	Consumption of unpasteurized cow milk (Yes; No); Consumption of cheese from unpasteurized cow milk (Yes; No)	0. No; 1. Yes	Variable for analysis compiled from the two questionnaire items
Consumption of unpasteurized sheep milk or cheese	Consumption of unpasteurized sheep milk (Yes; No); Consumption of cheese from unpasteurized sheep milk (Yes; No)	0. No; 1. Yes	Variable for analysis compiled from the two questionnaire items
Consumption of unpasteurized goat milk or cheese	Consumption of unpasteurized goat milk (Yes; No); Consumption of cheese from unpasteurized goat milk (Yes; No)	0. No; 1. Yes	Variable for analysis compiled from the two questionnaire items
Contact with dog	Yes; No	0. No; 1. Yes	
Contact with cat	Yes; No	0. No; 1. Yes	
Found ticks on domestic animal	How often ticks found on dog (number/week); How often ticks found on cat (number/week); How often ticks found on other household animal (number/week),	0. No; 1. Yes	Variable for analysis compiled from the three questionnaire items.
Reported exposure to tick	Yes/No	0. No; 1. Yes	
Known place of exposure to ticks	Known place (Yes; No); Name of closest town (free text); Longitude and Latitude from map	0. No; 1. Yes	
Used insect repellent on clothes	never, sometimes, always	0. No; 1. Yes	
Wear long pants outdoors	never, sometimes, always	0. No; 1. Yes	
Tuck pants legs into socks	never, sometimes, always	0. No; 1. Yes	
Check self for tick back home	never, sometimes, always	0. No; 1. Yes	

Place of residence was classified by endemic or non-endemic areas, according to the official definition that the average incidence in each administrative district did or did not exceed 1 case per 100,000 inhabitants in the preceding 5-year period (for more information, see Supplementary Table S1, and Supplementary Figures S1 and S2, online material). We stratified the analysis according to endemic and non-endemic status of study subjects’ residence for two reasons. First, residence in endemic region modified the effects of other variables on TBE risk in preliminary analyses in the entire dataset. Secondly, the existence of infected ticks arises from persistent enzootic cycles, due to environmental and biological, rather than human, factors. This stratification was therefore considered because many of the factors potentially associated with infection within endemic regions would not necessarily pre-dispose people to infection in non-endemic regions.

### Multivariate Model

Conditional logistic regression was used to account for the matched study design. A stepwise and backwards selection model-building strategy was first used to create intermediate models for each of the following groups of factors: socio-economic factors, residence characteristics, travel history, outdoor exposures. In the case of travel history, destinations within TBE-endemic or non-endemic regions were distinguished, and the duration of the travel during the exposure period was determined. Initially, the factors significant at p≤0.1 level in the univariate analysis were considered, and then factors significant in the intermediate models were further included in an initial full multivariate model. In the multivariate model we assessed confounding by each of the candidate variables by inspecting the impact of its inclusion/exclusion on the estimates of the effect of the remaining variables. If time spent at different outdoor locations was identified as a significant risk factor (p≤0.05), the relative importance of occupational or recreational exposures was examined and related to specific activities. We considered two-way interactions between spending ≥10 hours/week of recreational time in locations significantly associated with TBE risk and specific recreational activities. Education and occupation were considered only in adults. We checked the adequacy of the model using the Pregibon goodness-of-link test. This test re-runs the conditional logistic regression on the predicted logit score and its square, and the interpretation is based on the significance of the square term. As a sensitivity test, we also re-ran the model with and without children and major occupational groups.

The effect of each ordinal variable (education category, income category, distance of the residence from the woods, duration of exposure time) was considered as a categorical as well as a scored variable, including linear and higher order terms. Categories that showed <20% effect, and were not significantly different by the Wald test, were grouped. The most meaningful variable form was selected based on information criteria (Bayesian (BIC) and Akaike (AIC) - see Supplementary Tables S4, S11) and transparency of interpretation. The robustness of model parameters was assessed by their sensitivity to excluding defined population groups (children, occupational groups). No significant impact of these procedures on the model parameters was noted.

Adjusted population attributable fractions (PAF) were estimated for selected variables by the method of Bruzzi et al. (1985) [36], for which the primary underlying assumption is that the cases could be considered a random sample of those in the population. The variables were selected according to their significance in univariate and multivariate analyses and their relevance to public health. Bootstrap standard errors and bias-corrected (BC) confidence intervals were estimated for the adjusted population attributed risks. The bootstrap program first repeated the conditional logistic regression on each sample, and then estimated PAFs on that sample. We generated 5,024 completed bootstrap samples for the endemic area analysis and 9,984 samples for the non-endemic analysis.

All analyses were conducted in STATA versions 10 and 12 (StataCorp, College Station, Texas, USA).

## Results

### Study Population Characteristics

The outcome of the recruitment process, including validation of the matching procedures, is summarized in Figure 1. In total, 178 matched pairs were used for the analysis, including one (33 pairs), two (142 pairs), three (2 pairs), and four (1 pair) controls per case, making a total of 505 valid interviews. Of 178 cases, 145 (81%) met criteria for confirmed cases, and the remaining 33 cases were confirmed by high concentrations of IgM anti-TBEV antibodies in serum. The comparative characteristics of the two study sub-populations (Table 2) confirm the good match between cases and controls. The mean period between the TBE onset and interview among cases was 28.9 days (SE 2.0 days). The equivalent among respective controls was 58.3 days (SE 2.6).

**Table 2:** Demographic characteristics of studied subjects, by endemic status of their residence, national case-control study of TBE risk factors, Poland, January–December 2009.

Characteristic	Endemic regions			Non-endemic regions		
	Cases (%)n = 124	Controls (%)n = 222	p-value*	Cases (%)n = 54	Controls (%)n = 105	p-value*
**Age (years)**			0.856			0.987
<20	14 (11.3)	30 (13.5)		10 (18.5)	17 (16.2)	
20–29	22 (17.7)	30 (13.5)		9 (16.7)	15 (14.3)	
30–39	14 (11.3)	25 (11.3)		8 (14.8)	18 (17.1)	
40–49	20 (16.1)	41 (18.5)		9 (16.7)	16 (15.2)	
50–59	36 (29.0)	69 (31.1)		10 (18.5)	23 (21.9)	
>60	18 (14.5)	27 (12.2)		8 (14.8)	16 (15.2)	
**Gender**			0.905			0.990
Males	40 (32.3)	73 (32.9)		20 (37.0)	39 (37.1)	
Females	84 (67.7)	149 (67.1)		34 (64.0)	66 (62.9)	
**Urbanization**			0.967			0.664
Rural	78 (62.9)	142 (64.0)		30 (55.6)	66 (62.9	
City <100,000	44 (35.5)	77 (34.7)		14 (25.9)	22 (21.0)	
City ≥100,000	2 (1.6)	3 (1.4)		10 (18.5)	17 (16.2)	

* Comparison of distribution of matched variables between cases and controls, chi-square test.

### Risk Factors in TBE Endemic Areas

The univariate associations between all the studied factors and TBE risk among inhabitants of endemic areas (summarized in Table 3, with complete information in Supplementary Table S2) indicate the importance of socio-economic characteristics. First, risk of TBE decreased with the increasing education level, and with increasing income per household member, but this trend was not statistically significant. Secondly, non-specialized occupational groups (technicians and associate professionals, craft and related trade workers and elementary occupations), foresters and the unemployed, in that order, were characterized by increasingly strong risk of symptomatic TBE infection compared with other employment groups. People who lived further from the nearest forest suffered significantly lower TBE risk. While travel to (other) endemic regions had no effect, travel to non-endemic areas (i.e. out of an endemic area) during the exposure period had a significant protective effect, showing a dose response with respect to duration (Supplementary Table S7).

**Table 3:** Univariate and multivariate associations between the studied variables and TBE risk among inhabitants of endemic areas, Poland, 2009.

Studied variable/category	Cases (%) n = 124	Controls (%) n = 222	Univariate analyses					Multivariate analysis				
			OR	95% CI		p-value*		aOR	95% CI		p-value	
**Education level**												
Child <16 years old	8 (6.5)	20 (9.1)	ref.				**0.023**					
Primary/vocational	80 (64.5)	109 (49.5)	3.88	0.39–38.24								
High school or higher	36 (29.0)	91 (41.4)	2.11	0.20–21.84								
**Income per household member (US dollars)** †												
<160	46 (37.1)	78 (35.1)	ref.				0.456					
160–320	51 (41.1)	85 (38.3)	0.96		0.57–1.61							
320–480	21 (16.9)	39 (17.6)	0.90		0.45–1.78							
>480	6 (4.8)	20 (9.0)	0.47		0.17–1.27							
**Occupation**												
Technical, craft & elementary occupations	29 (24.6)	35 (15.8)‡	2.73		1.39–5.37			5.39	2.21–13.16		**<0.001**	
Forestry workers	7 (5.9)	4 (1.8)‡	4.34		1.21–15.56			8.96	1.58–50.77		**0.013**	
Unemployed	14 (11.9)	8 (3.6)‡	5.34		1.94–14.68			11.51	2.84–46.59		**0.001**	
Other status (including students and retired)	68 (57.6)	174 (78.7)‡	ref.				**<0.001**					
**Distance from residence to nearest forest**												
≤500 m	70 (56.9)	101 (45.5)	ref.				**0.067**	ref.				
>500 m	53 (43.1)	121 (54.5)	0.67		0.43–1.03			0.44	0.24–0.80		**0.007**	
**Travel history**												
In-country travel to endemic region	20 (16.1)	45 (20.5)	0.76		0.42–1.36		0.350					
In-country travel to non-endemic region	12 (9.7)	39 (17.7)	0.49		0.24–0.97		**0.034**	0.38	0.15–0.93		**0.034**	
**Occupational time spent outdoors in each habitat (≥10 hours/week ** ***vs*** ** less)**												
Deciduous forests	3 (2.4)	2 (0.9)	2.30		0.38–14.12		0.361					
Coniferous forest	4 (3.2)	4 (1.8)	1.69		0.42–6.85		0.467					
Mixed forests	8 (6.5)	7 (3.2)	2.21		0.76–6.44		0.145					
Forest edges	4 (3.2)	16 (7.2)	0.38		0.12–1.19		**0.074**	0.14	0.03–0.55		**0.005**	
Meadows/high grass	6 (4.8)	23 (10.4)	0.42		0.15–1.18		**0.078**					
Town parks/city streets	2 (1.6)	8 (3.6)	0.40		0.08–1.98		0.227					
Cottage gardens	2 (1.6)	2 (0.9)	1.41		0.19–10.34		0.733					
Fields/farms	9 (7.3)	30 (13.5)	0.49		0.22–1.10		**0.072**					
**Recreational time spent outdoors in each habitat (≥10 hours/week ** ***vs*** ** less)**												
Deciduous forests	0 (0.0)	4 (1.8)	–		–		–					
Coniferous forest	0 (0.0)	4 (1.8)	–		–		–					
Mixed forests	19 (15.3)	13 (5.9)	3.11		1.42–6.81		**0.004**	0.57**		0.07–4.57	0.598	
Forest edges	9 (7.3)	13 (5.9)	1.33		0.53–3.36		0.548					
Meadows/high grass	5 (4.0)	12 (5.4)	0.77		0.26–2.26		0.624					
Town parks/city streets	4 (3.2)	12 (5.4)	0.58		0.18–1.93		0.361					
Cottage gardens	9 (7.3)	21 (9.5)	0.80		0.34–1.90		0.612					
Fields/farms	4 (3.2)	7 (3.2)	0.85		0.22–3.31		0.818					
**Recreational outdoor activities (activity ** ***vs*** ** no activity)**												
Hunting	4 (3.2)	6 (2.7)	1.27		0.33–4.90		0.728					
Fishing	22 (17.7)	40 (18.0)	1.03		0.56–1.89		0.918					
Sailing	6 (4.8)	5 (2.3)	2.35		0.71–7,75		0.162					
Camping	7 (5.6)	37 (16.7)	0.25		0.09–0.66		**0.001**	0.17		0.05–0.61	**0.006**	
Hiking	55 (44.4)	101 (45.5)	0.94		0.60–1.47		0.789					
Cycling	52 (41.9)	99 (44.6)	0.88		0.54–1.43		0.609					
Gardening	81 (65.3)	145 (65.3)	1.00		0.61–1.65		1.000					
Swimming outdoors	19 (15.3)	53 (23.9)	0.47		0.23–0.94		**0.026**	0.24		0.09–0.61	**0.003**	
Collection of forest foods	71 (57.3)	104 (46.8)	1.50		0.92–2.44		0.100	1.29**		0.67–2.48	0.444	
Interaction - time spent recreationally in mixed forest and collecting foods								19.19		1.72–214.32	**0.016**	

Results from conditional logistic regression.

* p-value for the likelihood ratio (LR) chi-square test computed for the univariate statistics; Note: for ordinal variables this approximates a test for trend;

† calculated from local currency (PLN) as at January–December 2009;

‡ the denominator for percentages were non-missing observations;

** variables included in a significant interaction, therefore aOR must be interpreted together with the interaction term (final row); OR - odds ratio from univariate analyses; aOR - adjusted odds ratio for variables retained in the final multivariate model; CI – confidence interval.

Certain aspects of human activities had significant impacts on TBE risk (Table 3). Spending ≥10 hours per week in mixed forests in relation to either occupational or recreational activities was associated with increased risk of TBE (OR 2.21 and 3.11, respectively). As expected, lengthy occupational exposure in mixed forests was strongly associated with being a forester (data not shown), already identified as a significant risk factor. Somewhat paradoxically, occupational exposure of ≥10 hours per week at forest edges substantially decreased TBE risk. The particular types of recreational activity also proved to be relevant: spending time camping or swimming was associated with significantly reduced risk for TBE, whereas collecting forests foods and sailing was associated with increased risk (Table 3).

Based on univariate analysis and intermediate models (Supplementary Tables S5, S6, S7, S8, S9), the following candidate variables were considered in the final model: education (high school or higher; primary/vocational), occupation (technicians, craftsmen and elementary occupations; forestry or fishery workers; unemployed; others), distance from residence to forest (≤500 m vs >500 m), travel to non-endemic area during exposure period (yes/no), ≥10 h/week spent in mixed forest in relation to work (yes/no), ≥10 h/week spent in mixed forest during leisure activities (yes/no), ≥10 h/week spent at forest edge in relation to work (yes/no), sailing (yes/no), camping (yes/no), collecting mushrooms/berries (yes/no), swimming outdoors (yes/no) (Supplementary Table S10).

**Figure 2:**
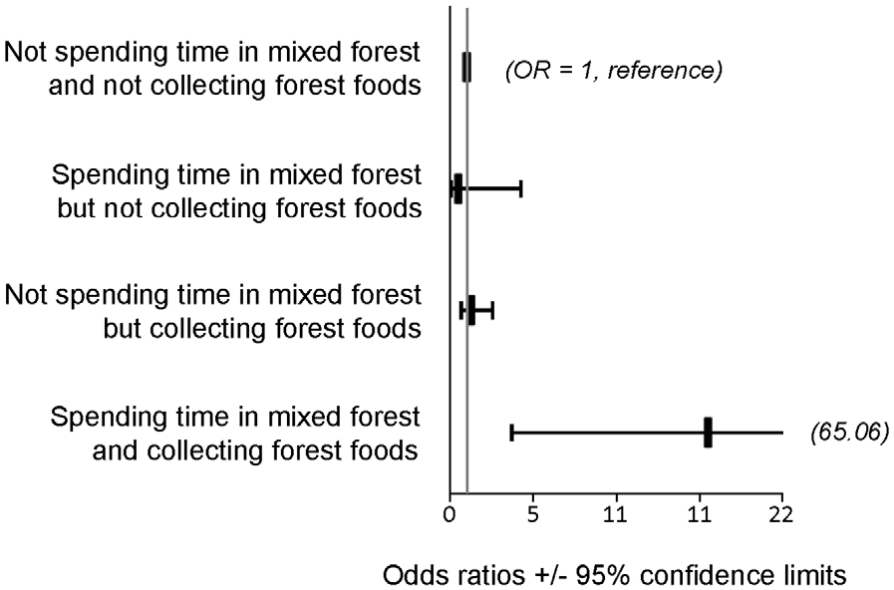
Interaction between time spent in mixed forest and collecting forest foods. Odds ratios and 95% confidence intervals, TBE case-control study, Poland, January–December 2009.

Of the socio-economic factors, occupation remained a strong predictor of TBE, with the unemployed, foresters, and non-specialized occupations the most affected (aOR 11.51, 8.96 and 5.39 respectively) (Table 3). The effect of working at forest edges ≥10 h/week was significantly protective in the final model (aOR 0.14), but this term may include being outside the forest and highlights the much lesser risk compared with working within deciduous or mixed forests. After adjusting for socio-demographic and outdoor exposures, camping and swimming remained protective (aOR 0.17 and 0.24, respectively). Neither recreation for ≥10 hours per week in mixed forests nor collecting forest foods (mushrooms or berries) *per se* was a high-risk activity, but the combination of these two activities conferred the highest risk for TBE (aOR 19.19, see also Figure 2).

### Risk Factors in TBE Non-endemic Areas

No socio-economic factors predicted TBE risk among inhabitants of non-endemic areas, although there was a hint of a protective effect of higher education in the univariate analysis (Table 4, complete information in Supplementary Table S3). Curiously, residence at a greater distance from the nearest forest was associated with increasing risk, whereas travel to (other) non-endemic areas reduced the risk. Amongst outdoor recreational activities, exposure of at least 10 hours per week in mixed forests was a significant risk factor, while equivalent time spent in cottage gardens was a strong protective factor. No specific recreational activity was associated with TBE risk.

**Table 4:** Univariate and multivariate association between studied variables and the TBE risk among inhabitants of non-endemic areas, results from conditional logistic regression, Poland, 2009.

Studied variable/category	Cases (%)n = 54	Controls (%)n = 105	Univariate analyses			Multivariate analysis		
			OR	95% CI	p-value*	aOR	95% CI	p-value
**Education level**								
Child <16 years old	2 (3.8)‡	3 (2.9)‡	ref.		0.536			
Primary/vocational	32 (61.5)‡	56 (54.4)‡	0.50	0.03–7.99				
High school or higher	18 (34.6)‡	44 (42.7)‡	0.34	0.02–5.99				
**Income per household member (US dollars)** †								
<160	17 (31.5)	28 (26.7)	Ref		0.703			
160–320	21 (38.9)	47 (44.8)	0.73	0.30–1.77				
320–480	11 (20.4)	17 (16.2)	0.88	0.30–2.58				
>480	5 (9.3)	13 (12.4)	0.49	0.12–2.07				
**Occupation**								
Technical, craft & elementary occupations	12 (22.2)‡	21 (20.0)‡	1.39	0.54–3.58				
Forestry workers	3 (5.6)‡	1 (1.0)‡	6.36	0.60–67.25				
Unemployed	3 (5.6)‡	9 (8.6)‡	0.57	0.11–2.99				
Other status (including students and retired)	35 (64.8)‡	72 (68.6)‡	ref.		0.326			
**Distance from residence to nearest forest**								
≤500 m	21 (38.9)	57 (54.3)	ref.		**0.044**			
>500 m	33 (61.1)	48 (45.7)	2.28	1.01–5.15		4.00	1.49–10.75	**0.006**
**Travel history**								
In-country travel to endemic region	4 (7.4)	2 (1.9)	3.61	0.65–19.91	0.126	4.65	0.59–36.50	0.144
In-country travel to non-endemic region	8 (14.8)	29 (27.6)	0.40	0.16–1.01	**0.037**	0.33	0.12–0.94	**0.038**
**Occupational time spent outdoors in each habitat (≥10 hours/week ** ***vs*** ** less)**								
Deciduous forests	4 (7.4)	4 (3.8)	2.26	0.49–10.42	0.295			
Coniferous forest	5 (9.3)	5 (4.8)	2.19	0.58–8.36	0.250			
Mixed forests	4 (7.4)	5 (4.8)	2.00	0.40–9.91	0.401			
Forest edges	3 (5.6)	7 (6.7)	0.72	0.14–3.74	0.686			
Meadows/high grass	2 (3.7)	10 (9.5)	0.35	0.07–1.73	0.159			
Town parks/city streets	0 (0.0)	5 (4.8)						
Cottage gardens	0 (0.0)	2 (1.9)						
Fields/farms	4 (7.4)	9 (8.6)	0.60	0.15–2.34	0.447			
**Recreational time spent outdoors in each habitat (≥10 hours/week ** ***vs*** ** less)**								
Deciduous forests	4 (7.4)	4 (3.8)	2.26	0.49–10.42	0.295			
Coniferous forest	3 (5.6)	3 (2.9)	2.38	0.38–14.97	0.351			
Mixed forests	12 (22.2)	7 (6.7)	4.95	1.56–15.69	**0.003**	7.18	1.90–27.08	**0.004**
Forest edges	10 (18.5)	9 (8.6)	3.65	1.09–12.23	**0.029**			
Meadows/high grass	7 (13.0)	19 (18.1)	0.58	0.17–1.99	0.384			
Town parks/city streets	7 (13.0)	20 (19.0)	0.57	0.19–1.72	0.309			
Cottage gardens	2 (3.7)	20 (19.0)	0.18	0.04–0.78	**0.005**			
Fields/farms	8 (14.8)	16 (15.2)	1.18	0.38–3.68	0.783			
**Recreational outdoor activities (activity ** ***vs*** ** no activity)**								
Hunting	2 (3.7)	1 (1.0)	3.24	0.29–36.63	0.325			
Fishing	7 (13.0)	10 (9.5)	1.36	0.46–4.05	0.576			
Sailing	1 (1.9)	4 (3.8)	0.43	0.05–3.87	0.413			
Camping	5 (9.3)	7 (6.7)	1.51	0.38–5.96	0.559			
Hiking	20 (37.0)	48 (45.7)	0.63	0.31–1.29	0.202			
Cycling	29 (53.7)	60 (57.1)	0.89	0.44–1.77	0.733			
Gardening	33 (61.1)	70 (66.7)	0.78	0.37–1.64	0.512			
Swimming outdoors	7 (13.0)	17 (16.2)	0.64	0.23–1.81	0.390			
Collection of forest foods	26 (48.1)	51 (48.6)	1.00	0.48–2.05	0.995			

* p-value for the likelihood ratio (LR) chi-square test computed for the univariate statistics; Note: for ordinal variables this approximates a test for trend;

† calculated from local currency (PLN) as at January-December 2009;

‡ the denominator for percentages were non-missing observations; OR - odds ratio from univariate analyses; aOR - adjusted odds ratio for variables retained in the final multivariate model; CI – confidence interval.

Based on the univariate analysis and the intermediate models (Supplementary Tables S12, S13, S14, S15, S16), the following candidate variables were included in the initial full multivariate model: education (per one level increase), occupation (forester vs other), residence distance from the forest (≤500 m vs >500 m), travel to non-endemic area (yes/no), travel to endemic area (yes/no), ≥10 h/week in mixed forest during leisure time (yes/no), ≥10 h/week in cottage gardens (yes/no) (Supplementary Table S17). In the final model (Table 4), spending ≥10 h/week in mixed forest during leisure time was the single most important predictor of TBE risk (aOR 7.18). After adjusting for socio-demographic variables and outdoor exposures, the effect of increasing distance between residence and forests remained significant (aOR 4.00). A history of travel by inhabitants of non-endemic areas to endemic areas returned a high adjusted odds ratio (aOR 4.65), but this was non-significant. Conversely, travel to non-endemic areas was significantly associated with decreased risk, even after adjusting for other factors (aOR 0.33).

### Estimation of Population Attributable Fraction

**Figure 3:**
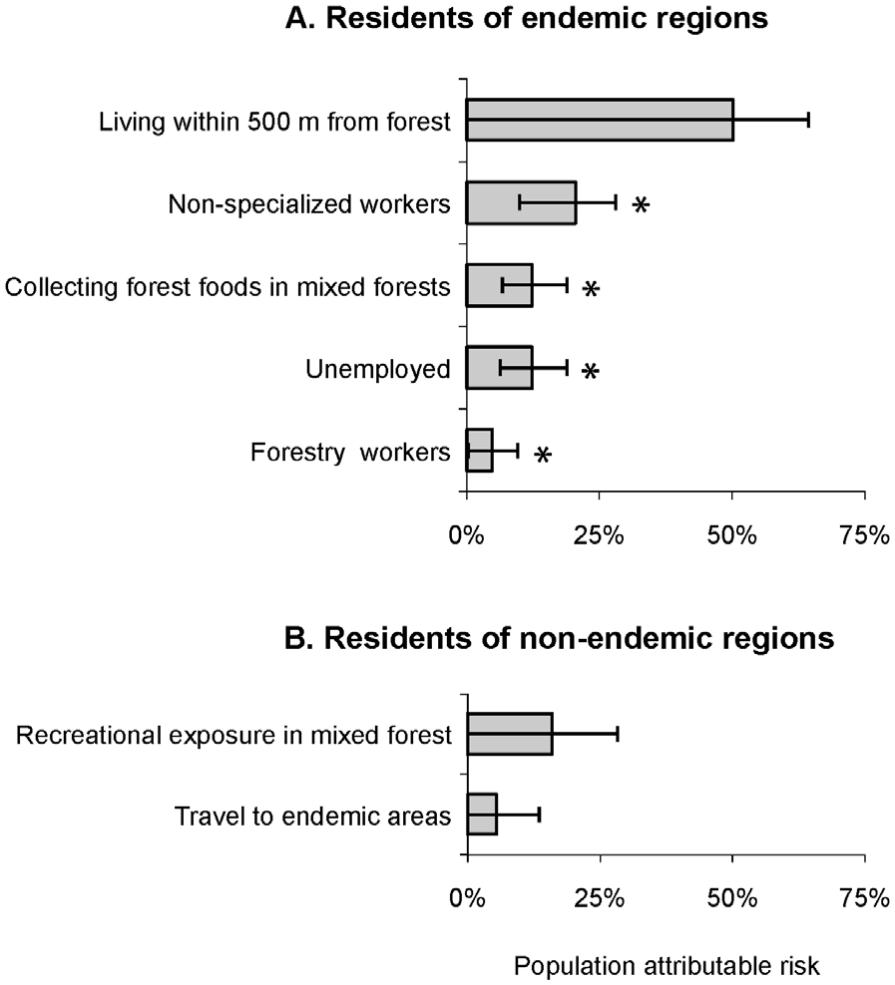
Estimates of population attributable fraction. Selected risk factors and 95% confidence intervals, TBE case-control study, Poland, January–December 2009. * indicates statistically significant effects (p<0.05).

Among inhabitants of endemic areas, population attributable fraction (PAF) was established for persons living within 500 m of a forest (0.312), the occupational groups of technicians, craftsman and elementary workers (0.202), unemployed (0.109) and foresters (0.053), and persons who spent ≥10 hours of recreation per week collecting forest foods in mixed forests (0.127) (Figure 3A). All effects, apart from distance from home to the nearest forest, were statistically significant. Among inhabitants of non-endemic areas, PAFs were established for persons spending ≥10 hours of recreation per week in mixed forests (0.191), and travelling to endemic areas (0.058) (Figure 3B). Only the former effect, however, was statistically significant in non-endemic areas.

## Discussion

Despite many constraints in ascertaining behavioural exposure of humans to ticks, and in measuring many factors that have important influences on TBE risk (such as weather conditions and populations of wild animals and ticks within the disease foci), this first case-control study of individual TBE risk factors allows deeper insight into human behaviour and characteristics that increase the risk of contacting ticks infected with TBEV. In endemic areas, highest TBE risk was associated with recreation of ≥10 hours/week in mixed forests and harvesting forest foods, being unemployed, or employed as a forester or non-specialized worker. Outside TBE endemic areas, risk was greater for those who spent ≥10 hours/week on recreation in mixed forests and visited known TBE endemic areas. This result, derived from the first rigorous epidemiological study for TBE in Europe, establishes the principal that human factors do play a role in determining risk of infection, and therefore could have been instrumental in driving the recent increases in incidence, despite assertions to the contrary [17], [18]. The particular patterns of these effects will vary between countries.

### Public Health Implications of Main Results

The findings identify certain sections of the population at highest risk of TBE infection, allowing public health interventions to be targeted more effectively and efficiently. Two methods were applied: using conditional logistic regression, we identified risk factors amongst the (sampled) population as a whole; then, based on the PAF calculation, we assessed the proportion of cases that would be avoided if the risk factor were eliminated from the population (for example by immunization of the risk groups). The combined results enable prioritization of possible interventions that could have the highest impact on TBE incidence in Poland. The importance of lower socio-economic status in determining risk highlights the mis-match between greatest need and least capacity to implement protection without financial assistance.

First there is the environmental context of zoonotic risk. As expected, mixed forests were identified as significant places of human exposure associated with TBE risk, as these habitats provide the most favourable abiotic conditions for ticks [37] and house abundant tick hosts. Secondly, there is human exposure to zoonotic hazard. It is well known that forestry work poses a high risk most probably related not only to the time spent in the forested ecosystems, but also to the types of activity, for example frequently leaving paths and moving amongst the vegetation and so enhancing contact with ticks. Even so, due to universal vaccination of forestry workers provided freely by forestry departments during the previous decade, the effect of forestry-related occupational exposure is likely to be underestimated. The limited system of recording vaccine use in Poland does not take into account the type of vaccination (primary or booster), and therefore does not permit a valid estimation of vaccination coverage in the general population and in the groups of foresters [34]. According to the information obtained in the State Forest Directorate, no-cost vaccination is offered to all employees, but its use is not recorded at national level. The estimated national immunization coverage for Poland in 2007 was 0.8% [34]. With respect to recreational exposure associated with mixed forests, in the case of residents in non-endemic regions travel to endemic regions was a necessary additional risk factor, while staying within non-endemic regions (or travel there by residents of endemic regions) not surprisingly reduced risk. Within endemic regions, residence close to forests has a high positive impact on TBE risk, as was also found for Lyme borreliosis in Pennsylvania, USA [38], presumably simply reflecting the probability of entering tick-infested forests. The finding that, conversely, risk of symptomatic TBE infection is higher for residents living further from forests in non-endemic regions is hard to explain without invoking the possibility of reduced protection (e.g. barrier clothing, vaccination) due to lower awareness of risk, for which we have no evidence. Basing vaccination policies solely on the propinquity of homes to forests, therefore, would be neither specific nor sensitive enough, given the other risk factors and the contrast between residents of endemic and non-endemic areas.

Amongst the range of outdoor activities examined, collecting forest foods (mushrooms or berries) *per se* did not increase risk unless it occurred in mixed forests, when it became the highest identified risk factor. This finding concurs with individual responses to questionnaires in a survey in Latvia [35], [39] that revealed that collecting forest foods was the commonest reason for frequent visits to forests (more often than once a month) and also more than doubled the odds of suffering a tick bite, second only to forestry work. In contrast, camping and swimming in Poland were strongly negatively associated with TBE risk, presumably because such activities occupied people away from tick habitats, as apparently did prolonged recreation in cottage gardens in non-endemic areas. To conclude, TBE risk seems to be related not to time spent outdoors *per se*, but to specific activities that lead people to maximum exposure to specific vegetation where TBEV-infected ticks are present.

Compared with previous ecological studies that identified socio-economic correlates of behaviour associated with TBE risk (e.g. frequent visits to forests principally for food harvest in Latvia) [35], the socio-economic factors examined here can be related directly to individual TBE risk. There was no statistically significant protective effect of increasing income and education level, but occupation appears as a particularly important risk determinant, although only in endemic areas, as would be expected. In addition to forestry, unemployment and the group of non-specialized occupations are unambiguously associated with higher risk.

This strong empiric evidence for unemployment and relatively lowly paid work as important contributing factors for public health problems (see also [40], [41], [42], [43]) is backed by several plausible mechanisms in relation to this particular infectious disease that have already been substantiated with respect to high risk behaviour in Latvia [35] and the unemployment-triggered spike in TBE cases in Lithuania, Latvia and Poland in 2009 [30].

First, harvesting food from forests, although by no means practiced only by people of low economic status, was the major reason given for frequent visits to forests by the unemployed in Latvia. Poland is Europe’s leading exporter of wild fungi. A nation-wide survey performed in Poland in 2004 found that the harvest of these and other forest foods to generate additional family income is associated with low income, and worsening of financial situation was given as a major reason for increased harvest by less wealthy families [44], [45]. Podlaskie is the most productive province for mushrooms, followed by Warminsko-Mazurskie [46], both of which suffer particularly high TBE incidence. Officially recorded annual harvests in Poland were more variable for forest foods than for game animals, as would be expected from weather effects on productivity. Harvests in 2009 were typical for the past decade: mushrooms (principally chanterelle, boletus and king boletus), 4,176 tonnes (range of annual harvests 2,379–6,922); fruits and nuts (principally bilberry, elder, dog rose and mountain ash), 12,244 tonnes (range 8,374–19,138); game, 7,147 tonnes (range 6,549–9,546) (http://www.stat.gov.pl/cps/rde/xbcr/gus/PUBL_sy_statistical_yearbook_agriculture_2011.pdf). In Russia, of the workers who moved out of employment on the traditional collective and state farms, and then out of the corporate farms that succeeded them after 1990, more than half shifted to individual employment on household plots and peasant farms; sale of mushrooms and forest fruits made up two-thirds of the income from non-farm self-employment amongst these rural people [45]. In Lithuania in 2009, when unemployment increased after the downward trend of previous years, the official market in wild fungi doubled (http://www.stat.gov.lt).

Secondly, unemployment may render people unable to cover the cost of the vaccine, or even the cost of tick repellents. Indeed, increasing costs and decreasing uptake of vaccination were recorded in Lithuania during the recent recession [30].

Thirdly, if unemployment were associated with a lower standard of living, including lower levels of nutrition, protective immune responses against infection might be compromised, leading to more severe clinical symptoms and thus a higher proportion of infections progressing to recorded neuro-invasive disease (see below), as stressful life events can have an impact on the health of an individual, including immunological health, acting through stress hormones [47], [48]. It should be noted, however, that improved wealth and the funding of relatively high-cost leisure activities in rural settings may also increase the risk of TBE, as appears more likely to apply in the Czech Republic [19]. This conforms to the conceptual model that both poverty and wealth affect zoonotic risk [25], but asymmetrically due to differential constraints and opportunities for amelioration [49].

The case-control study reported here allows appropriate responses by national public health agencies to geographically variable risk factors, both within and between countries. A full relative cost-benefit analysis is needed, including all realistic logistical and practical aspects, to decide between the strategies of encouraging the lower cost but less secure use of tick repellants and protective clothing *versus* the higher cost but much more certain protection of vaccination. Individual perception of risk and personal attitudes towards vaccination, depending on geographical and social contexts, also needs further systematic study.

### Study Limitations

As with all observational studies, our study has several limitations. In Poland, testing for TBE is limited to the cases with symptoms of meningo-encephalitis, representing approximately 5% of persons exposed to TBE virus, because most infections remain asymptomatic, and 70% of symptomatic infections are limited to the first, flu-like phase without progressing to CNS involvement [3]. This study therefore does not reflect risk factors for TBEV infection, but rather for development of severe neuro-invasive disease. Non-compliance to study participation might introduce bias, but only if it were differential with respect to disease status. This disease carries no stigma in Poland, but controls and less debilitated patients, more occupied by work, might have been less available or willing to devote time to the interview. Interviewers, however, were trained to accommodate this in the times they sought contact with subjects and arranged interviews.

The possibility of having included as controls persons who had recently suffered an asymptomatic TBE infection could have added noise to the results. This effect could be more pronounced in endemic compared to non-endemic regions, due to higher prevalence to TBE-infected ticks. TBE infections, however, are relatively rare even if, in reality, there are 20 infections per reported case. Our study does, in any case, conform to the case-cohort study design by having selected members of the control group at random from the source population [50].

A potential problem of over-matching cases and controls with respect to socio-economic class arises if socially deprived and relatively wealthy people occupy spatially distinct areas. To minimize this effect, the selected geographical units within which cases and controls were matched were relatively large, inhabited on average by 100,000 persons (NUTS-4 administrative area). To accommodate the low incidence but extensive distribution of TBE in Poland, 90 interviewers had to be recruited, but they were drawn as much as possible from amongst health department surveillance epidemiologists with extensive experience of interviewing communicable disease patients. They were trained and equipped to maximize the accuracy of subjects recalling events up to six weeks prior to the interview (see methods). The number of questions that required interpretation by the interviewer was limited and the use of *aide-memoires* followed a strict protocol.

Finally, the problem of confounding variables of known and unknown origin was minimized as far as possible by the careful handling of the data. Case and control subjects were matched on potentially strong confounders (age, gender and district of residence), and potential confounders were included in the multivariable analysis. The variable concerning time spent travelling to non-endemic areas (i.e. while not in endemic areas), for example, corrected for the time that did not contribute to the relevant exposure period.

### Conclusions

Despite the potential for bias and confounding, our study design allowed a more accurate insight into individual-level risk factors for TBE in Poland than from recent ecologic-type studies. Its methodological strength lies with random selection of control subjects from the general population and rigorous procedures to avoid recall bias. Gratifyingly, the results from both study types were largely concordant, thereby validating many of the substantive conclusions on determinants of TBE risk in central and eastern European countries. It is increasingly clear that human factors must be taken into account in assessing and therefore combating emerging zoonotic risk. Such factors can change adversely more rapidly than environmental conditions, but are also more amenable to public health measures. There is no reason to think that these general conclusions would not apply to other countries, but the specific risk factors are likely to vary with differing national cultural and socio-economic contexts and can only be identified with certainty by focused case-control studies. In wealthier countries, for example, or those where harvest of forest foods is not a strong cultural tradition, there is unlikely to be such a strong association of unemployment or low-paid work with exposure through activities in tick-infested forests. Instead, the scaling of risk with economic hardship is likely to be reversed [49].

## Supporting Information

Figure S1 **Map of Polish provinces included in the study, with definition of TBE endemic area.**
(DOCX)

Figure S2 **Map of TBE endemic areas and numbers of cases recruited to the study.**
(DOCX)

Table S1 **Number of TBE cases reported in Poland, by province of residence, and provinces included in the study, Poland, 2004–2008.**
(DOCX)

Table S2 **Univariate analysis in the subgroup of residents of endemic areas.**
(DOCX)

Table S3 **Univariate analysis in the subgroup of residents of non-endemic areas.**
(DOCX)

Table S4 **Selection of the form of ordered variables (endemic areas).**
(DOCX)

Table S5 **Effect of main socio-economic factors in endemic regions.**
(DOCX)

Table S6 **Main socio-economic factors in endemic regions.**
(DOCX)

Table S7 **Effect of place of residence and travel history in endemic regions.**
(DOCX)

Table S8 **Independent effects of total time spent at different locations and (for selected variables) in relation to work or recreation during exposure period in endemic regions.**
(DOCX)

Table S9 **Independent effects of spending leisure time in particular outdoor activities during exposure period in endemic region.**
(DOCX)

Table S10 **Model containing main effects for the candidate variables – endemic area.**
(DOCX)

Table S11 **Selection of the form of ordered variables (non-endemic areas).**
(DOCX)

Table S12 **Effect of main socio-economic factors in non-endemic regions.**
(DOCX)

Table S13 **Main socio-economic factors in non-endemic regions – proposed grouping of certain occupation variables.**
(DOCX)

Table S14 **Effect of place of residence and travel history in non-endemic regions.**
(DOCX)

Table S15 **Independent effects of total time spent at different locations and (for selected variables) in relation to work or recreation during exposure period in non-endemic regions.**
(DOCX)

Table S16 **Independent effects of spending leisure time on particular outdoor activities during exposure period in non-endemic region.**
(DOCX)

Table S17 **Model containing main effects for the candidate variables in non-endemic areas.**
(DOCX)

Text S1 **Rationale for figures and tables below.**
(DOCX)
