# Supplementary material for: A National Case-Control Study Identifies Human Socio-Economic Status and Activities as Risk Factors for Tick-Borne Encephalitis in Poland
Source: PLoS One. 2012 Sep 19;7(9):e45511. doi: 10.1371/journal.pone.0045511 (PMC3446880; doi:10.1371/journal.pone.0045511)
Supplement: Figure S1 — Map of Polish provinces included in the study, with definition of TBE endemic area. (DOCX) [file pone.0045511.s001.docx]

**Figure S1**. Polish provinces with names, with the definition of an endemic area. Provinces included in the study are shaded, Poland, 2004-2008.


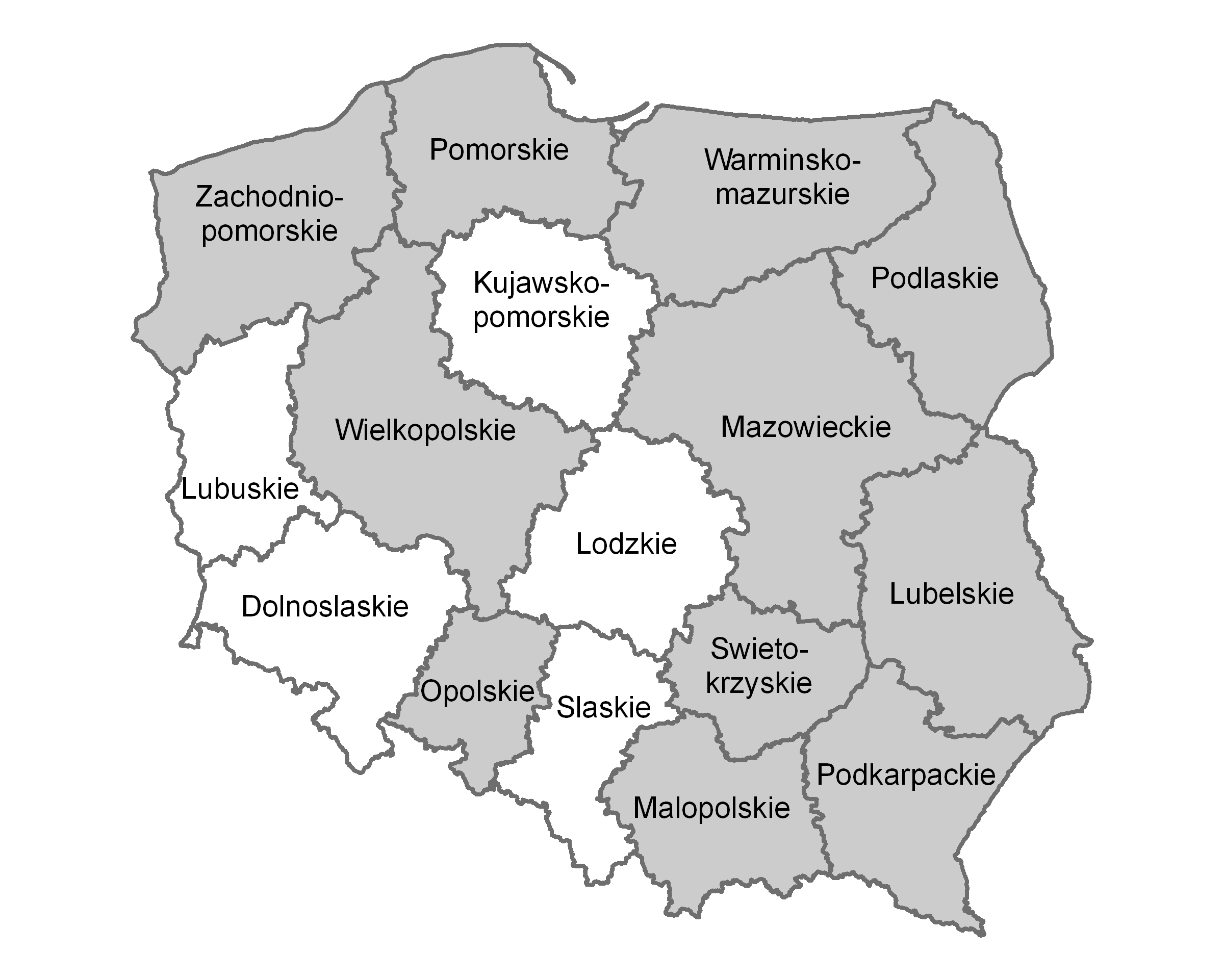


**Definition of endemic area**

An endemic area for tick-borne encephalitis is defined as an administrative district (poviat) in which the average incidence of locally-exposed tick-borne encephalitis exceeds 1 case per 100,000 inhabitants during the previous five years. Township districts are assigned the category of the surrounding rural districts.

*NOTE: The above definition is currently used for research purposes only. It is planned to implement a relevant definition of endemic areas by the national public health authorities in order to improve the targeting of vaccination recommendations.*
