# Supplementary material for: A National Case-Control Study Identifies Human Socio-Economic Status and Activities as Risk Factors for Tick-Borne Encephalitis in Poland
Source: PLoS One. 2012 Sep 19;7(9):e45511. doi: 10.1371/journal.pone.0045511 (PMC3446880; doi:10.1371/journal.pone.0045511)

**Figure S2**. Location of TBE endemic areas and the number of cases recruited to the study by administrative district (NUTS-4), Poland 2009.


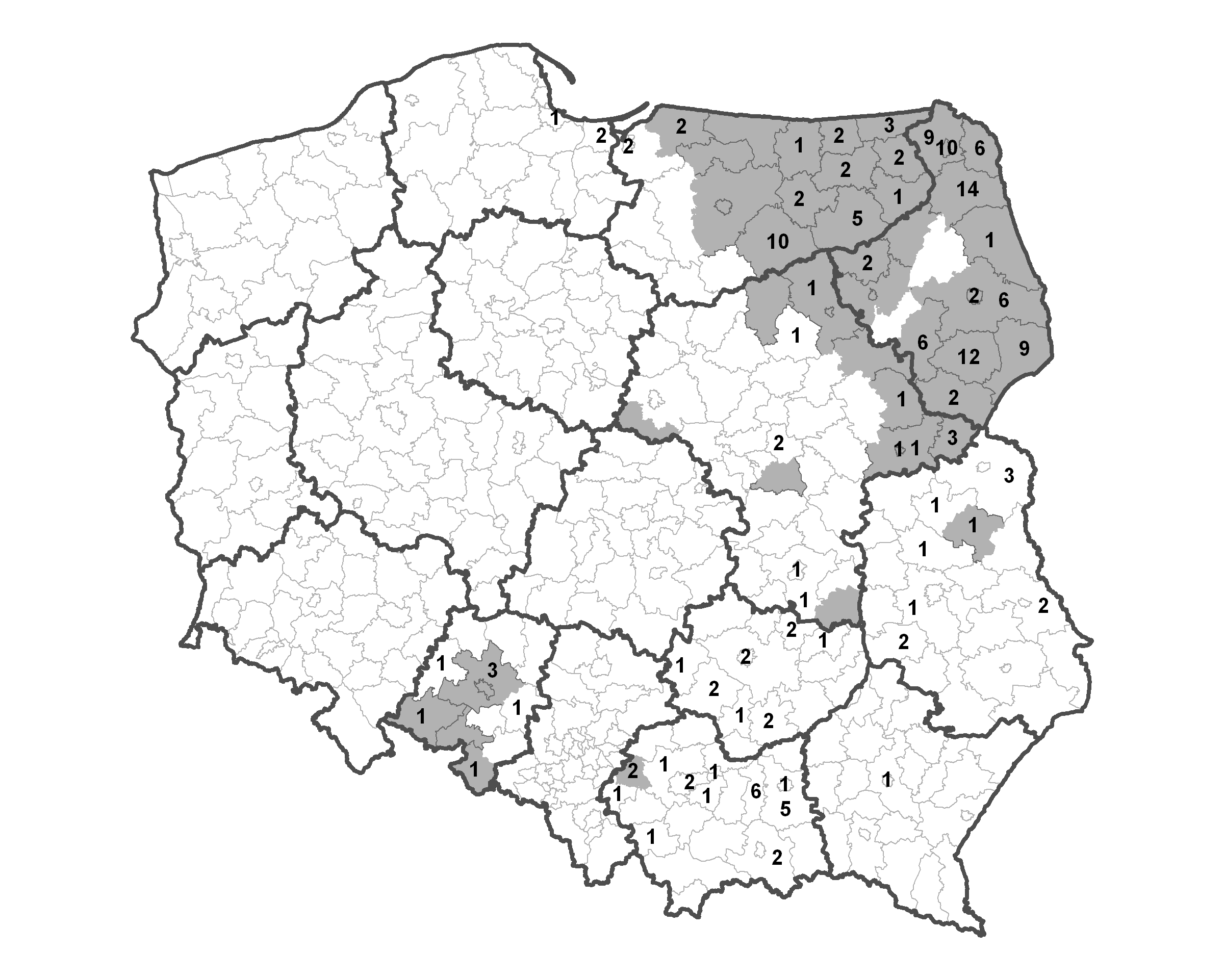

Supplement: Figure S2 — Map of TBE endemic areas and numbers of cases recruited to the study. (DOCX) [file pone.0045511.s002.docx]
