# Supplementary material for: A National Case-Control Study Identifies Human Socio-Economic Status and Activities as Risk Factors for Tick-Borne Encephalitis in Poland
Source: PLoS One. 2012 Sep 19;7(9):e45511. doi: 10.1371/journal.pone.0045511 (PMC3446880; doi:10.1371/journal.pone.0045511)
Supplement: Table S1 — Number of TBE cases reported in Poland, by province of residence, and provinces included in the study, Poland, 2004–2008. (DOCX) [file pone.0045511.s003.docx]

**Table S1. Number of TBE cases reported in Poland, by province of residence, and provinces included in the study, Poland, 2004-2008.**

| **Province** | **2004** | **2005** | **2006** | **2007** | **2008** | **5-year average** | **Included in 2009 study** |
| --- | --- | --- | --- | --- | --- | --- | --- |
| Dolnoslaskie | 3 | 6 | 1 | 0 | 2 | 2 | No |
| Kujawsko-pomorskie | 0 | 0 | 0 | 0 | 1 | 0 | No |
| Lubelskie | 3 | 2 | 5 | 4 | 2 | 3 | Yes* |
| Lubuskie | 0 | 0 | 0 | 0 | 0 | 0 | No |
| Lodzkie | 4 | 3 | 3 | 0 | 3 | 3 | No |
| Malopolskie | 1 | 0 | 8 | 15 | 11 | 7 | Yes |
| Mazowieckie | 27 | 6 | 28 | 18 | 14 | 19 | Yes |
| Opolskie | 3 | 15 | 12 | 2 | 11 | 9 | Yes |
| Podkarpackie | 1 | 0 | 1 | 0 | 2 | 1 | Yes* |
| Podlaskie | 113 | 94 | 155 | 99 | 97 | 112 | Yes |
| Pomorskie | 0 | 0 | 1 | 0 | 0 | 0 | Yes* |
| Slaskie | 3 | 0 | 5 | 2 | 2 | 2 | No |
| Swietokrzyskie | 1 | 4 | 7 | 1 | 3 | 3 | Yes* |
| Warminsko-mazurskie | 103 | 47 | 90 | 91 | 53 | 77 | Yes |
| Wielkopolskie | 0 | 0 | 0 | 1 | 0 | 0 | No |
| Zachodniopomorskie | 0 | 0 | 1 | 0 | 1 | 0 | Yes* |
| **POLAND** | **262** | **177** | **317** | **233** | **202** | **238** | **10** |

* Provinces included despite low incidence due to a parallel study during which all patients with aseptic CNS infection were tested for TBE (unpublished results)
