# Supplementary material for: A National Case-Control Study Identifies Human Socio-Economic Status and Activities as Risk Factors for Tick-Borne Encephalitis in Poland
Source: PLoS One. 2012 Sep 19;7(9):e45511. doi: 10.1371/journal.pone.0045511 (PMC3446880; doi:10.1371/journal.pone.0045511)
Supplement: Table S2 — Univariate analysis in the subgroup of residents of endemic areas. (DOCX) [file pone.0045511.s004.docx]

**Table S2**. **Univariate analysis in the subgroup of residents of endemic areas**

| **Variable** | **Cases** | | **Controls** | | **OR** | | **95% CI** | | **p-value** | |
| --- | --- | --- | --- | --- | --- | --- | --- | --- | --- | --- |
|  | **N (%)*** | | **N (%)*** | |  | |  | |  | |
| Education: | |  | |  | |  | |  | | **0.070** |
| (Child <16 years) | | 8 (6.5) | | 20 (9.1) | | 0.26 | | 0.03-2.56 | |  |
| Primary | | 40 (32.3) | | 57 (25.9) | | ref. | |  | |  |
| High school | | 27 (21.8) | | 59 (26.8) | | 0.64 | | 0.33-1.25 | |  |
| Vocational | | 40 (32.2) | | 52 (23.6) | | 1.05 | | 0.54-2.05 | |  |
| University | | 9 (7.3) | | 32 (14.6) | | 0.42 | | 0.17-1.04 | |  |
|  | |  | |  | |  | |  | |  |
| Income per family member, PLN (USD) | |  | |  | |  | |  | | 0.456 |
| <500 (<160) | | 46 (37.1) | | 78 (35.1) | | ref. | |  | |  |
| 500-1000 (160 - 320) | | 51 (41.1) | | 85 (38.3) | | 0.96 | | 0.57-1.61 | |  |
| 1000-1500 (320 – 480) | | 21 (17.0) | | 39 (17.6 ) | | 0.90 | | 0.45-1.78 | |  |
| >1500 (>480) | | 6 (4.8) | | 20 (9.0) | | 0.47 | | 0.17-1.27 | |  |
|  | |  | |  | |  | |  | |  |
| Occupation: | |  | |  | |  | |  | | **0.009** |
| Child | | 8 (6.8) | | 20 (9.1) | | 0.87 | | 0.03-22.76 | |  |
| Students | | 6 (5.1) | | 15 (6.8) | | 0.82 | | 0.09-7.26 | |  |
| Managers | | 0 (0) | | 8 (3.6) | | - | | - | |  |
| Professionals | | 2 (1.7) | | 6 (2.7) | | 0.89 | | 0.12-6.78 | |  |
| Technicians and associate professionals | | 10 (8.5) | | 13 (5.9) | | 2.87 | | 0.67-12.34 | |  |
| Clerical support workers | | 2 (1.7) | | 10 (4.5) | | 0.56 | | 0.07-4.27 | |  |
| Service and sales workers | | 4 (3.4) | | 6 (2.7) | | 1.80 | | 0.33-9.66 | |  |
| Agricultural workers | | 21 (17.8) | | 59 (26.7) | | 0.94 | | 0.31-2.85 | |  |
| Forestry or fishery workers† | | 7 (5.9) | | 4 (1.8) | | 4.29 | | 0.83-22.23 | |  |
| Craft and related trades workers | | 10 (8.5) | | 14 (6.3) | | 2.61 | | 0.66-10.35 | |  |
| Plant and machine operators, and assemblers | | 7 (5.9) | | 15 (6.8) | | 1.65 | | 0.36-7.44 | |  |
| Elementary occupations | | 9 (7.6) | | 8 (3.6) | | 3.01 | | 0.69-13.01 | |  |
| Unemployed | | 14 (11.9) | | 8 (3.6) | | 5.39 | | 1.30-22.37 | |  |
| Retired | | 18 (15.2) | | 35 (15.9) | | ref. | |  | |  |
|  | |  | |  | |  | |  | |  |
| Forest proximity (from place of residence) | |  | |  | |  | |  | | **0.043** |
| <50 m | | 22 (17.9) | | 22 (9.9) | | ref. | |  | |  |
| 50-100 m | | 23 (18.7) | | 27 (12.2) | | 0.90 | | 0.40-2.04 | |  |
| 100-500 m | | 25 (20.3) | | 52 (23.4) | | 0.51 | | 0.24-1.09 | |  |
| 500-1000 m | | 23 (18.7) | | 35 (15.8) | | 0.75 | | 0.35-1.63 | |  |
| >1 km | | 30 (24.4) | | 86 (38.7) | | 0.41 | | 0.20-0.82 | |  |
|  | |  | |  | |  | |  | |  |
| Living on a farm | |  | |  | |  | |  | | 0.541 |
| No | | 74 (59.7) | | 125 (56.3) | | ref. | |  | |  |
| Yes | | 50 (40.3) | | 97 (43.7) | | 0.86 | | 0.54-1.38 | |  |
|  | |  | |  | |  | |  | |  |
| Goats on the farm | |  | |  | |  | |  | |  |
| No | | 12 (26.7) | | 25 (28.7) | | ref. | |  | | 0.909 |
| Yes | | 33 (73.3) | | 61 (71.3) | | 0.92 | | 0.24-3.55 | |  |
|  | |  | |  | |  | |  | |  |
| Sheep on the farm | |  | |  | |  | |  | | - |
| No | | 41 (100) | | 83 (98.8) | | - | | - | |  |
| Yes | | 0 (0) | | 1 (1.2) | | - | | - | |  |
|  | |  | |  | |  | |  | |  |
| Cows on the farm | |  | |  | |  | |  | | - |
| No | | 41 (100) | | 81 (96.4) | | - | | - | |  |
| Yes | | 0 (0) | | 3 (3.6) | | - | | - | |  |
|  | |  | |  | |  | |  | |  |
| Living in a house with a yard/garden | |  | |  | |  | |  | | 0.289 |
| No | | 20 (16.1) | | 28 (12.6) | | ref. | |  | |  |
| Yes | | 104 (83.9) | | 194 (87.4) | | 0.70 | | 0.36-1.36 | |  |
|  | |  | |  | |  | |  | |  |
| Yard/garden secured from wild animals | |  | |  | |  | |  | | 0.250 |
| No | | 29 (27.9) | | 41 (21.2) | | ref. | |  | |  |
| Yes | | 75 (72.1) | | 152 (78.8) | | 0.72 | | 0.41-1.26 | |  |
|  | |  | |  | |  | |  | |  |
| Wild animals ever seen in the yard/garden | |  | |  | |  | |  | | **0.072** |
| No | | 94 (90.4) | | 159 (82.0) | | ref. | |  | |  |
| Yes | | 10 (9.6) | | 35 (18.0) | | 0.52 | | 0.25-1.09 | |  |
|  | |  | |  | |  | |  | |  |
| Travel history | |  | |  | |  | |  | |  |
| In-country travel to endemic region | |  | |  | |  | |  | | 0.350 |
| No | | 104 (83.9) | | 175 (79.6) | | ref. | |  | |  |
| Yes | | 20 (16.1) | | 45 (20.4) | | 0.76 | | 0.42-1.36 | |  |
|  | |  | |  | |  | |  | |  |
| In country travel to non-endemic region | |  | |  | |  | |  | | **0.034** |
| No | | 112 (90.3) | | 181 (82.3) | | ref. | |  | |  |
| Yes | | 12 (9.7) | | 39 (17.7) | | 0.49 | | 0.24-0.97 | |  |
|  | |  | |  | |  | |  | |  |
| Time spent travelling during the exposure period | |  | |  | |  | |  | |  |
| no travel | | 93 (75.0) | | 149 (68.7) | | ref. | |  | | **0.068** |
| < 5 days | | 23 (18.6) | | 42 (19.4) | | 0.88 | | 0.49-1.59 | |  |
| 5 - 15 days | | 3 (2.4) | | 18 (8.3) | | 0.19 | | 0.04-0.84 | |  |
| ≥15 days | | 5 (4.0) | | 8 (3.7) | | 0.74 | | 0.17-3.19 | |  |
|  | |  | |  | |  | |  | |  |
| Time spent travelling to endemic areas during the exposure period | |  | |  | |  | |  | |  |
| no travel | | 104 (83.9) | | 176 (80.7) | | ref. | |  | | 0.706 |
| <5 days | | 16 (12.9) | | 33 (15.2) | | 0.78 | | 0.41-1.50 | |  |
| ≥5 days | | 4 (3.2) | | 9 (4.1) | | 0.78 | | 0.23-2.68 | |  |
|  | |  | |  | |  | |  | |  |
| Time spent travelling to non-endemic areas during the exposure period | |  | |  | |  | |  | |  |
| no travel | | 109 (87.9) | | 174 (79.1) | | ref. | |  | | **0.052** |
| <5 days | | 11 (8.9) | | 29 (13.2) | | 0.58 | | 0.27-1.24 | |  |
| ≥5 days | | 4 (3.2) | | 17 (7.7) | | 0.34 | | 0.11-1.05 | |  |
|  | |  | |  | |  | |  | |  |
| Travel distance | |  | |  | |  | |  | | 0.287 |
| Near residence <50 km or no travel | | 97 (79.5) | | 156 (72.2) | | ref. | |  | |  |
| ≥50km travel to endemic region | | 15 (12.3) | | 34 (15.7) | | 0.64 | | 0.32-1.30 | |  |
| ≥50km travel to non-endemic region only | | 10 (8.2) | | 26 (12.1) | | 0.62 | | 0.28-1.40 | |  |
|  | |  | |  | |  | |  | |  |
| Travel abroad | |  | |  | |  | |  | | 0.841 |
| No | | 119 (96.0) | | 214 (96.4) | | ref. | |  | |  |
| Yes | | 5 (4.0) | | 8 (3.6) | | 1.13 | | 0.35-3.65 | |  |
|  | |  | |  | |  | |  | |  |
| Time spent outdoors | |  | |  | |  | |  | | 0.803 |
| <20 hours per week | | 34 (27.4) | | 59 (26.7) | | ref. | |  | |  |
| 20-40 hours per week | | 49 (39.5) | | 78 (35.3) | | 1.02 | | 0.55-1.90 | |  |
| 40-60 hours per week | | 28 (22.6) | | 60 (27.1) | | 0.76 | | 0.38-1.56 | |  |
| >60 hours per week | | 13 (10.5) | | 24 (10.9) | | 0.83 | | 0.36-1.95 | |  |
|  | |  | |  | |  | |  | |  |
| Time spent outdoors in relation to work | |  | |  | |  | |  | | 0.393 |
| 0h per week | | 68 (54.8) | | 109 (49.1) | | ref. | |  | |  |
| 1-10h per week | | 10 (8.1) | | 14 (6.3) | | 0.95 | | 0.39-2.32 | |  |
| 11-20h per week | | 7 (5.7) | | 13 (5.9) | | 0.85 | | 0.32-2.28 | |  |
| 21-30h per week | | 3 (2.4) | | 12 (5.4) | | 0.31 | | 0.08-1.22 | |  |
| 31-40h per week | | 10 (8.0) | | 14 (6.3) | | 1.04 | | 0.43-2.52 | |  |
| >40h per week | | 26 (21.0) | | 60 (27.0) | | 0.60 | | 0.31-1.15 | |  |
|  | |  | |  | |  | |  | |  |
| Leisure time spent outdoors | |  | |  | |  | |  | | 0.372 |
| 0h per week | | 18 (14.5) | | 49 (22.1) | | ref. | |  | |  |
| 1-10h per week | | 25 (20.2) | | 45 (20.2) | | 1.50 | | 0.75-3.00 | |  |
| 11-20h per week | | 30 (24.2) | | 43 (19.4) | | 1.94 | | 0.90-4.19 | |  |
| 21-30h per week | | 15 (12.1) | | 33 (14.9) | | 1.30 | | 0.56-3.00 | |  |
| 31-40h per week | | 36 (29.0) | | 52 (23.4) | | 1.95 | | 0.91-4.19 | |  |
| >40h per week | |  | |  | |  | |  | |  |
|  | |  | |  | |  | |  | |  |
| Outdoor activities (activity vs no activity) | |  | |  | |  | |  | |  |
| Hunting | |  | |  | |  | |  | | 0.728 |
| No | | 120 (96.8) | | 216 (97.3) | | ref. | |  | |  |
| Yes | | 4 (3.2) | | 6 (2.7) | | 1.27 | | 0.33-4.90 | |  |
|  | |  | |  | |  | |  | |  |
| Camping | |  | |  | |  | |  | | **0.001** |
| No | | 117 (94.3) | | 185 (83.3) | | ref. | |  | |  |
| Yes | | 7 (5.7) | | 37 (16.7) | | 0.25 | | 0.09-0.66 | |  |
|  | |  | |  | |  | |  | |  |
| Fishing | |  | |  | |  | |  | | 0.918 |
| No | | 102 (82.3) | | 182 (82.0) | | ref. | |  | |  |
| Yes | | 22 (17.7) | | 40 (18.0) | | 1.03 | | 0.56-1.89 | |  |
|  | |  | |  | |  | |  | |  |
| Swimming outdoors (natural waters) | |  | |  | |  | |  | | **0.026** |
| No | | 105 (84.7) | | 169 (76.1) | | ref. | |  | |  |
| Yes | | 19 (15.3) | | 53 (23.9) | | 0.47 | | 0.23-0.94 | |  |
|  | |  | |  | |  | |  | |  |
| Sailing | |  | |  | |  | |  | | 0.162 |
| No | | 118 (95.2) | | 217 (97.8) | | ref. | |  | |  |
| Yes | | 6 (4.8) | | 5 (2.2) | | 2.35 | | 0.71-7.75 | |  |
|  | |  | |  | |  | |  | |  |
| Hiking | |  | |  | |  | |  | | 0.789 |
| No | | 69 (55.6) | | 121 (54.5) | | ref. | |  | |  |
| Yes | | 55 (44.4) | | 101 (45.5) | | 0.94 | | 0.60-1.47 | |  |
|  | |  | |  | |  | |  | |  |
| Cycling | |  | |  | |  | |  | | 0.609 |
| No | | 72 (58.1) | | 123 (55.4) | | ref. | |  | |  |
| Yes | | 52 (41.9) | | 99 (44.6) | | 0.88 | | 0.54-1.43 | |  |
|  | |  | |  | |  | |  | |  |
| Mushroom/berries collecting | |  | |  | |  | |  | | 0.100 |
| No | | 53 (42.7) | | 118 (53.2) | | ref. | |  | |  |
| Yes | | 71 (57.3) | | 104 (46.9) | | 1.50 | | 0.92-2.44 | |  |
|  | |  | |  | |  | |  | |  |
| Gardening | |  | |  | |  | |  | | 1.000 |
| No | | 43 (34.7) | | 77 (34.7) | | ref. | |  | |  |
| Yes | | 81 (65.3) | | 145 (65.3) | | 1.00 | | 0.61-1.65 | |  |
|  | |  | |  | |  | |  | |  |
| Place of outdoor leisure (>10 h/week) | |  | |  | |  | |  | |  |
| Deciduous forests | |  | |  | |  | |  | |  |
| No | | 124 (100) | | 218 (98.2) | | . | |  | |  |
| Yes | | 0 (0) | | 4 (1.8) | | . | |  | |  |
|  | |  | |  | |  | |  | |  |
| Coniferous forest | |  | |  | |  | |  | |  |
| No | | 124 (100) | | 218 (98.2) | | . | |  | |  |
| Yes | | 0 (0) | | 4 (1.8) | | . | |  | |  |
|  | |  | |  | |  | |  | |  |
| Mixed forests | |  | |  | |  | |  | | **0.004** |
| No | | 105 (84.7) | | 209 (94.1) | | ref. | |  | |  |
| Yes | | 19 (15.3) | | 13 (5.9) | | 3.11 | | 1.42-6.81 | |  |
|  | |  | |  | |  | |  | |  |
| Forest edges | |  | |  | |  | |  | | 0.548 |
| No | | 115 (92.7) | | 209 (94.1) | | ref. | |  | |  |
| Yes | | 9 (7.3) | | 13 (5.9) | | 1.33 | | 0.53-3.36 | |  |
|  | |  | |  | |  | |  | |  |
| Meadows/high grass | |  | |  | |  | |  | | 0.624 |
| No | | 119 (96.0) | | 210 (94.6) | | ref. | |  | |  |
| Yes | | 5 (4.0) | | 12 (5.4) | | 0.77 | | 0.26-2.26 | |  |
|  | |  | |  | |  | |  | |  |
| Town parks | |  | |  | |  | |  | |  |
| Yes | | 123 (99.2) | | 218 (98.2) | | ref. | |  | |  |
| No | | 1 (0.8) | | 4 (1.8) | | 0.43 | | 0.05-3.87 | | 0.413 |
|  | |  | |  | |  | |  | |  |
| City streets | |  | |  | |  | |  | |  |
| No | | 121 (97.6) | | 213 (96.0) | | ref. | |  | |  |
| Yes | | 3 (2.4) | | 9 (4.0) | | 0.60 | | 0.16-2.25 | | 0.433 |
|  | |  | |  | |  | |  | |  |
| Cottage gardens | |  | |  | |  | |  | |  |
| No | | 115 (92.7) | | 201 (90.5) | | ref. | |  | |  |
| Yes | | 9 (7.3) | | 21 (9.5) | | 0.80 | | 0.34-1.90 | | 0.612 |
|  | |  | |  | |  | |  | |  |
| Fields / farms | |  | |  | |  | |  | |  |
| No | | 120 (96.8) | | 215 (96.8) | | ref. | |  | |  |
| Yes | | 4 (3.2) | | 7 (3.2) | | 0.85 | | 0.22-3.31 | | 0.818 |
|  | |  | |  | |  | |  | |  |
| Place of work time spent outdoors (>10 h/week) | |  | |  | |  | |  | |  |
| Deciduous forests | |  | |  | |  | |  | |  |
| No | | 120 (97.6) | | 220 (99.1) | | ref. | |  | |  |
| Yes | | 3 (2.4) | | 2 (0.9) | | 2.30 | | 0.38-14.12 | | 0.361 |
|  | |  | |  | |  | |  | |  |
| Coniferous forest | |  | |  | |  | |  | |  |
| No | | 119 (96.8) | | 218 (98.2) | | ref. | |  | |  |
| Yes | | 4 (3.2) | | 4 (1.8) | | 1.69 | | 0.42-6.85 | | 0.467 |
|  | |  | |  | |  | |  | |  |
| Mixed forests | |  | |  | |  | |  | |  |
| No | | 115 (93.5) | | 215 (96.8) | | ref. | |  | |  |
| Yes | | 8 (6.5) | | 7 (3.2) | | 2.21 | | 0.76-6.44 | | 0.145 |
|  | |  | |  | |  | |  | |  |
| Forest edges | |  | |  | |  | |  | |  |
| No | | 119 (96.7) | | 206 (92.8) | | ref. | |  | |  |
| Yes | | 4 (3.3) | | 16 (7.2) | | 0.38 | | 0.12-1.19 | | **0.074** |
|  | |  | |  | |  | |  | |  |
| Meadows/high grass | |  | |  | |  | |  | |  |
| No | | 117 (95.1) | | 199 (89.6) | | ref. | |  | |  |
| Yes | | 6 (4.89) | | 23 (10.4) | | 0.42 | | 0.15-1.18 | | **0.078** |
|  | |  | |  | |  | |  | |  |
| Town parks | |  | |  | |  | |  | |  |
| No | | 123 (100) | | 221 (99.6) | | . | |  | |  |
| Yes | | 0 (0) | | 1 (0.4) | | . | |  | |  |
|  | |  | |  | |  | |  | |  |
| City streets | |  | |  | |  | |  | |  |
| No | | 121 (98.4) | | 215 (96.8) | | ref. | |  | |  |
| Yes | | 2 (1.6) | | 7 (3.2) | | 0.51 | | 0.09-2.77 | | 0.421 |
|  | |  | |  | |  | |  | |  |
| Cottage gardens | |  | |  | |  | |  | |  |
| No | | 121 (98.4) | | 220 (99.1) | | ref. | |  | |  |
| Yes | | 2 (1.6) | | 2 (0.9) | | 1.41 | | 0.19-10.34 | | 0.733 |
|  | |  | |  | |  | |  | |  |
| Fields / farms | |  | |  | |  | |  | |  |
| No | | 114 (92.7) | | 192 (86.5) | | ref. | |  | |  |
| Yes | | 9 (7.3) | | 30 (13.5) | | 0.49 | | 0.22-1.10 | | **0.072** |
|  | |  | |  | |  | |  | |  |
|  | |  | |  | |  | |  | |  |
| Place of total time spent outdoors (>10 h/week) | |  | |  | |  | |  | |  |
| No | | 121 (97.6) | | 215 (97.3) | | ref. | |  | |  |
| Yes | | 3 (2.4) | | 6 (2.7) | | 0.78 | | 0.19-3.18 | | 0.726 |
|  | |  | |  | |  | |  | |  |
| Coniferous forest | |  | |  | |  | |  | |  |
| No | | 120 (96.8) | | 213 (96.4) | | ref. | |  | |  |
| Yes | | 4 (3.2) | | 8 (3.6) | | 0.87 | | 0.25-3.08 | | 0.832 |
|  | |  | |  | |  | |  | |  |
| Mixed forests | |  | |  | |  | |  | |  |
| No | | 99 (79.8) | | 203 (91.9) | | ref. | |  | |  |
| Yes | | 25 (20.2) | | 18 (8.1) | | 2.84 | | 1.45-5.54 | | **0.002** |
|  | |  | |  | |  | |  | |  |
| Forest edges | |  | |  | |  | |  | |  |
| No | | 111 (89.5) | | 195 (88.2) | | ref. | |  | |  |
| Yes | | 13 (10.5) | | 26 (11.8) | | 0.85 | | 0.40-1.82 | | 0.680 |
|  | |  | |  | |  | |  | |  |
| Meadows/high grass | |  | |  | |  | |  | |  |
| No | | 113 (91.1) | | 187 (84.6) | | ref. | |  | |  |
| Yes | | 11 (8.9) | | 34 (15.4) | | 0.56 | | 0.26-1.18 | | 0.115 |
|  | |  | |  | |  | |  | |  |
| Town parks | |  | |  | |  | |  | |  |
| No | | 120 (96.8) | | 208 (94.1) | | ref. | |  | |  |
| Yes | | 4 (3.2) | | 13 (5.9) | | 0.54 | | 0.16-1.75 | | 0.283 |
|  | |  | |  | |  | |  | |  |
| City streets | |  | |  | |  | |  | |  |
| No | | 119 (96.0) | | 207 (93.7) | | ref. | |  | |  |
| Yes | | 5 (4.0) | | 14 (6.3) | | 0.63 | | 0.22-1.80 | | 0.374 |
|  | |  | |  | |  | |  | |  |
| Cottage gardens | |  | |  | |  | |  | |  |
| No | | 113 (91.1) | | 199 (90.0) | | ref. | |  | |  |
| Yes | | 11 (8.9) | | 22 (10.0) | | 0.92 | | 0.41-2.07 | | 0.836 |
|  | |  | |  | |  | |  | |  |
| Fields / farms | |  | |  | |  | |  | |  |
| No | | 112 (90.3) | | 184 (83.3) | | ref. | |  | |  |
| Yes | | 12 (9.7) | | 37 (16.7) | | 0.45 | | 0.21-0.98 | | **0.037** |
|  | |  | |  | |  | |  | |  |
| Place of outdoor exposure during leisure time | |  | |  | |  | |  | | **0.002** |
| <10h/week outdoors | | 18 (14.5) | | 49 (22.1) | | ref. | |  | |  |
| >10h/week outdoors but <10h/week in forest | | 87 (70.2) | | 154 (69.4) | | 1.42 | | 0.77-2.64 | |  |
| >10h/week in forest but <10h/week in mixed forest | | 0 (0) | | 6 (2.7) | | - | | - | |  |
| >10h/week in mixed forest | | 19 (15.3) | | 13 (5.8) | | 3.87 | | 1.58-9.51 | |  |
|  | |  | |  | |  | |  | |  |
| Place of outdoor exposure in relation to work | |  | |  | |  | |  | | **0.040** |
| <10h/week outdoors | | 78 (62.9) | | 123 (55.4) | | ref. | |  | |  |
| >10h/week outdoors but <10h/week in forest | | 33 (26.6) | | 89 (40.1) | | 0.57 | | 0.33-0.99 | |  |
| >10h/week in forest but <10h/week in mixed forest | | 5 (4.0) | | 3 (1.3) | | 2.21 | | 0.49-9.97 | |  |
| >10h/week in mixed forest | | 8 (6.5) | | 7 (3.2) | | 1.55 | | 0.53-4.49 | |  |
|  | |  | |  | |  | |  | |  |
| Place of outdoor exposure in total | |  | |  | |  | |  | | **0.020** |
| <10h/week outdoors | | 7 (5.7) | | 17 (7.7) | | ref. | |  | |  |
| >10h/week outdoors but <10h/week in forest | | 87 (70.2) | | 178 (80.2) | | 1.11 | | 0.40-3.08 | |  |
| >10h/week in forest but <10h/week in mixed forest | | 5 (4.0) | | 9 (4.0) | | 1.21 | | 0.27-5.45 | |  |
| >10h/week in mixed forest | | 25 (20.1) | | 18 (8.1) | | 3.11 | | 1.03-9.42 | |  |
|  | |  | |  | |  | |  | |  |
| Consumption of unpasteurized cow milk or cheese | |  | |  | |  | |  | |  |
| No | | 82 (66.1) | | 137 (62.0) | | ref. | |  | |  |
| Yes | | 42 (33.9) | | 84 (38.0) | | 0.84 | | 0.51-1.36 | | 0.471 |
|  | |  | |  | |  | |  | |  |
| Consumption of unpasteurized sheep milk or cheese | |  | |  | |  | |  | |  |
| No | | 124 (100) | | 221 (100) | | - | | - | |  |
| Yes | | 0 (0) | | 0 (0) | | - | | - | |  |
|  | |  | |  | |  | |  | |  |
| Consumption of unpasteurized goat milk or cheese | |  | |  | |  | |  | |  |
| No | | 121 (97.6) | | 215 (97.3) | | ref. | |  | |  |
| Yes | | 3 (2.4) | | 6 (2.7) | | 0.83 | | 0.19-3.63 | | 0.805 |
|  | |  | |  | |  | |  | |  |
| Exposure to animals and tick bites | |  | |  | |  | |  | |  |
| Contact with dog | |  | |  | |  | |  | |  |
| No | | 10 (9.9) | | 13 (6.8) | | ref. | |  | |  |
| Yes | | 91 (90.1) | | 177 (93.2) | | 0.49 | | 0.18-1.35 | | 0.167 |
|  | |  | |  | |  | |  | |  |
| Contact with cat | |  | |  | |  | |  | |  |
| No | | 44 (44.4) | | 81 (42.9) | | ref. | |  | |  |
| Yes | | 55 (55.6) | | 108 (57.1) | | 0.96 | | 0.55-1.67 | | 0.888 |
|  | |  | |  | |  | |  | |  |
| Found ticks on domestic animal | |  | |  | |  | |  | |  |
| No | | 59 (58.4) | | 99 (52.1) | | ref. | |  | |  |
| Yes | | 42 (41.6) | | 91 (47.9) | | 0.70 | | 0.41-1.18 | | 0.173 |
|  | |  | |  | |  | |  | |  |
| Reported exposure to tick bite | |  | |  | |  | |  | | **<0.001** |
| No | | 46 (37.1) | | 199 (90.0) | | ref. | |  | |  |
| Yes | | 78 (62.9) | | 22 (10.0) | | 28.00 | | 10.20-76.87 | |  |
|  | |  | |  | |  | |  | |  |
| Known place of exposure to ticks: | |  | |  | |  | |  | | **<0.001** |
| No exposure to ticks | | 46 (37.1) | | 199 (90.1) | | ref. | |  | |  |
| Near residence | | 75 (60.5) | | 20 (9.1) | | 20.29 | | 10.44-82.19 | |  |
| During travel to endemic region | | 3 (2.4) | | 1 (0.4) | | 12.34 | | 0.67-226.43 | |  |
| During travel to non endemic region | | 0 (0) | | 1 (0.45) | | - | | - | |  |

* column percentages calculated excluding missing observations; † there were only forestry workers in the studied population
