# Supplementary material for: A National Case-Control Study Identifies Human Socio-Economic Status and Activities as Risk Factors for Tick-Borne Encephalitis in Poland
Source: PLoS One. 2012 Sep 19;7(9):e45511. doi: 10.1371/journal.pone.0045511 (PMC3446880; doi:10.1371/journal.pone.0045511)
Supplement: Table S3 — Univariate analysis in the subgroup of residents of non-endemic areas. (DOCX) [file pone.0045511.s005.docx]

**Table S3**. **Univariate analysis in the subgroup of residents of non-endemic areas**

| **Variable** | **Cases** | **Controls** | **OR** | **95% CI** | **p-value** |
| --- | --- | --- | --- | --- | --- |
|  | **N (%)*** | **N (%)*** |  |  |  |
| Education: |  |  |  |  | 0.329 |
| (Child <16 years) | 2 (3.8) | 3 (2.9) | 2.00 | 0.13-31.98 |  |
| Primary | 15 (28.8) | 18 (17.5) | ref. |  |  |
| High school | 11 (21.2) | 25 (24.3) | 0.43 | 0.15-1.24 |  |
| Vocational | 17 (32.7) | 38 (36.9) | 0.42 | 0.16-1.15 |  |
| University | 7 (13.5) | 19 (18.4) | 0.26 | 0.06-1.14 |  |
|  |  |  |  |  |  |
| Income per family member, PLN (USD) |  |  |  |  | 0.703 |
| <500 (<160) | 17 (31.5) | 28 (26.7) | ref. |  |  |
| 500-1000 (160 - 320) | 21 (38.9) | 47 (44.7) | 0.73 | 0.30-1.77 |  |
| 1000-1500 (320 – 480) | 11 (20.4) | 17 (16.2) | 0.88 | 0.30-2.58 |  |
| >1500 (>480) | 5 (9.2) | 13 (12.4) | 0.49 | 0.12-2.07 |  |
|  |  |  |  |  |  |
| Occupation: |  |  |  |  | 0.147 |
| Child | 2 (3.8) | 3 (2.9) | 2.67 | 0.03-211.00 |  |
| Students | 4 (7.5) | 9 (8.7) | 1.33 | 0.05-39.16 |  |
| Managers | 0 (0) | 2 (1.9) | - | - |  |
| Professionals | 2 (3.8) | 8 (7.8) | 0.48 | 0.02-9.59 |  |
| Technicians and associate professionals | 4 (7.5) | 11 (10.7) | 0.91 | 0.09-9.51 |  |
| Clerical support workers | 4 (7.5) | 6 (5.8) | 4.87 | 0.24-100.16 |  |
| Service and sales workers | 3 (5.7) | 4 (3.9) | 3.30 | 0.11-101.84 |  |
| Agricultural workers | 5 (9.4) | 7 (6.8) | 2.73 | 0.35-21.37 |  |
| Forestry or fishery workers† | 3 (5.7) | 1 (1.0) | 14.09 | 0.55-362.95 |  |
| Craft and related trades workers | 5 (9.4) | 10 (9.7) | 3.18 | 0.24-41.29 |  |
| Plant and machine operators, and assemblers | 3 (5.7) | 4 (3.9) | 3.76 | 0.23-60.20 |  |
| Elementary occupations | 3 (5.7) | 0 (0) | - | - |  |
| Unemployed | 3 (5.7) | 9 (8.7) | 0.78 | 0.04-14.36 |  |
| Retired | 12 (22.6) | 29 (28.2) | ref. |  |  |
|  |  |  |  |  |  |
| Forest proximity (from place of residence) |  |  |  |  | 0.373 |
| <50 m | 2 (3.7) | 7 (6.7) | ref. |  |  |
| 50-100 m | 8 (14.8) | 19 (18.1) | 1.37 | 0.23-8.30 |  |
| 100-500 m | 11 (20.4) | 31 (29.5) | 1.26 | 0.23-7.00 |  |
| 500-1000 m | 13 (24.1) | 19 (18.1) | 3.05 | 0.55-17.01 |  |
| >1 km | 20 (37.0) | 29 (27.6) | 2.68 | 0.50-14.36 |  |
|  |  |  |  |  |  |
| Living on a farm |  |  |  |  | 0.496 |
| No | 33 (61.1) | 68 (64.8) | ref. |  |  |
| Yes | 21 (38.9) | 37 (35.2) | 1.33 | 0.59-3.01 |  |
|  |  |  |  |  |  |
| Goats on the farm |  |  |  |  | 0.889 |
| No | 12 (63.2) | 20 (58.8) | ref. |  |  |
| Yes | 7 (36.8) | 14 (41.2) | 0.89 | 0.17-4.60 |  |
|  |  |  |  |  |  |
| Sheep on the farm |  |  |  |  | 0.862 |
| No | 17 (89.5) | 31 (91.2) | ref. |  |  |
| Yes | 2 (10.5) | 3 (8.8) | 1.20 | 0.16-9.20 |  |
|  |  |  |  |  |  |
| Cows on the farm |  |  |  |  |  |
| No | 19 (100) | 31 (91.2) | - | - |  |
| Yes | 0 (0) | 3 (8.2) | - | - |  |
|  |  |  |  |  |  |
| Living in a house with a yard/garden |  |  |  |  | 0.121 |
| No | 13 (24.1) | 14 (13.3) | ref. |  |  |
| Yes | 41 (75.9) | 91 (86.7) | 0.49 | 0.19-1.22 |  |
|  |  |  |  |  |  |
| Yard/garden secured from wild animals |  |  |  |  | 0.809 |
| No | 10 (25.0) | 25 (27.8) | ref. |  |  |
| Yes | 30 (75.0) | 65 (72.2) | 1.11 | 0.48-2.56 |  |
|  |  |  |  |  |  |
| Wild animals ever seen in the yard/garden |  |  |  |  | 0.479 |
| No | 32 (76.2) | 70 (76.1) | ref. |  |  |
| Yes | 10 (23.8) | 22 (23.9) | 1.40 | 0.55-3.57 |  |
|  |  |  |  |  |  |
| Travel history |  |  |  |  |  |
| In-country travel to endemic region |  |  |  |  | 0.126 |
| No | 50 (92.6) | 101 (98.1) | ref. |  |  |
| Yes | 4 (7.4) | 2 (1.9) | 3.61 | 0.65-19.91 |  |
|  |  |  |  |  |  |
| In country travel to non-endemic region |  |  |  |  | **0.037** |
| No | 46 (85.2) | 74 (71.8) | ref. |  |  |
| Yes | 8 (14.8) | 29 (28.2) | 0.40 | 0.16-1.01 |  |
|  |  |  |  |  |  |
| Time spent travelling during the exposure period |  |  |  |  | 0.680 |
| no travel | 42 (77.8) | 72 (69.2) | ref. |  |  |
| < 5 days | 7 (13.0) | 20 (19.2) | 0.58 | 0.20-1.67 |  |
| 5 – 15 days | 3 (5.6) | 7 (6.7) | 0.74 | 0.19-2.89 |  |
| ≥15 days | 2 (3.7) | 5 (4.8) | 0.60 | 0.11-3.16 |  |
|  |  |  |  |  |  |
| Time spent travelling to endemic areas during the exposure period |  |  |  |  | 0.578 |
| no travel | 50 (92.6) | 100 (96.2) | ref. |  |  |
| <5 days | 2 (3.7 ) | 2 (1.9) | 2.26 | 0.31-16.67 |  |
| ≥5 days | 2 (3.7) | 2 (1.9) | 2.26 | 0.31-16.67 |  |
|  |  |  |  |  |  |
| Time spent travelling to non-endemic areas during the exposure period |  |  |  |  | 0.101 |
| no travel | 46 (85.2) | 74 (71.2) | ref. |  |  |
| <5 days | 6 (11.1) | 20 (19.2) | 0.46 | 0.15-1.43 |  |
| ≥5 days | 2 (3.7) | 10 (9.6) | 0.31 | 0.07-1.43 |  |
|  |  |  |  |  |  |
| Travel distance |  |  |  |  | 0.157 |
| Near residence <50 km or no travel | 36 (75.0) | 74 (74.0) | ref. |  |  |
| ≥50km travel to endemic region | 4 (8.3) | 2 (2.0) | 3.12 | 0.55-17.73 |  |
| ≥50km travel to non-endemic region only | 8 (16.7) | 24 (24.0) | 0.58 | 0.22-1.50 |  |
|  |  |  |  |  |  |
| Travel abroad |  |  |  |  | 0.288 |
| No | 53 (98.2) | 100 (95.2) | ref. |  |  |
| Yes | 1 (1.8) | 5 (4.8) | 0.35 | 0.04-3.02 |  |
|  |  |  |  |  |  |
| Time spent outdoors |  |  |  |  | 0.747 |
| <20 hours per week | 16 (30.8) | 34 (33.0) | ref. |  |  |
| 20-40 hours per week | 28 (53.8) | 50 (48.5) | 1.39 | 0.59-3.24 |  |
| 40-60 hours per week | 3 (5.8) | 9 (8.8) | 0.69 | 0.15-3.20 |  |
| >60 hours per week | 5 (9.6) | 10 (9.7) | 1.18 | 0.27-5.10 |  |
|  |  |  |  |  |  |
| Time spent outdoors in relation to work |  |  |  |  | 0.247 |
| 0h per week | 35 (64.8) | 73 (69.5) | ref. |  |  |
| 1-10h per week | 3 (5.6) | 8 (7.6) | 0.76 | 0.17-3.30 |  |
| 11-20h per week | 4 (7.4) | 2 (1.9) | 7.53 | 0.74-77.05 |  |
| 21-30h per week | 3 (5.6) | 3 (2.9) | 2.98 | 0.38-23.48 |  |
| 31-40h per week | 3 (5.6) | 2 (1.9) | 2.76 | 0.43-17.82 |  |
| >40h per week | 6 (11.0) | 17 (16.2) | 0.86 | 0.24-3.10 |  |
|  |  |  |  |  |  |
| Leisure time spent outdoors |  |  |  |  | 0.217 |
| 0h per week | 8 (14.8) | 13 (12.4) | ref. |  |  |
| 1-10h per week | 6 (11.1) | 23 (21.9) | 0.47 | 0.13-1.63 |  |
| 11-20h per week | 15 (27.8) | 19 (18.1) | 1.34 | 0.40-4.50 |  |
| 21-30h per week | 10 (18.5) | 13 (12.4) | 1.72 | 0.45-6.53 |  |
| 31-40h per week | 15 (27.8) | 37 (35.2) | 0.70 | 0.19-2.63 |  |
| >40h per week |  |  |  |  |  |
|  |  |  |  |  |  |
| Outdoor activities (activity vs no activity) |  |  |  |  |  |
| Hunting |  |  |  |  | 0.325 |
| No | 52 (96.3) | 104 (99.0) | ref. |  |  |
| Yes | 2 (3.7) | 1 (1.0) | 3.24 | 0.29-36.63 |  |
|  |  |  |  |  |  |
| Camping |  |  |  |  | 0.559 |
| No | 49 (90.7) | 98 (93.3) | ref. |  |  |
| Yes | 5 (9.3) | 7 (6.7) | 1.51 | 0.38-5.96 |  |
|  |  |  |  |  |  |
| Fishing |  |  |  |  | 0.576 |
| No | 47 (87.0) | 95 (90.5) | ref. |  |  |
| Yes | 7 (13.0) | 10 (9.5) | 1.36 | 0.46-4.05 |  |
|  |  |  |  |  |  |
| Swimming outdoors (natural waters) |  |  |  |  | 0.390 |
| No | 47 (87.0) | 88 (83.8) | ref. |  |  |
| Yes | 7 (13.0) | 17 (16.2) | 0.64 | 0.23-1.81 |  |
|  |  |  |  |  |  |
| Sailing |  |  |  |  | 0.413 |
| No | 53 (98.2) | 101 (96.2) | ref. |  |  |
| Yes | 1 (1.8) | 4 (3.8) | 0.43 | 0.05-3.87 |  |
|  |  |  |  |  |  |
| Hiking |  |  |  |  | 0.203 |
| No | 34 (63.0) | 57 (54.3) | ref. |  |  |
| Yes | 20 (37.0) | 48 (45.7) | 0.63 | 0.31-1.29 |  |
|  |  |  |  |  |  |
| Cycling |  |  |  |  | 0.733 |
| No | 25 (46.3) | 45 (42.9) | ref. |  |  |
| Yes | 29 (53.7) | 60 (57.1) | 0.89 | 0.44-1.77 |  |
|  |  |  |  |  |  |
| Mushroom/berries collecting |  |  |  |  | 0.995 |
| No | 28 (51.9) | 54 (51.4) | ref. |  |  |
| Yes | 26 (48.1) | 51 (48.6) | 1.00 | 0.48-2.05 |  |
|  |  |  |  |  |  |
| Gardening |  |  |  |  | 0.512 |
| No | 21 (38.9) | 35 (33.3) | ref. |  |  |
| Yes | 33 (61.1) | 70 (66.7) | 0.78 | 0.37-1.64 |  |
|  |  |  |  |  |  |
| Place of outdoor leisure (>10 h/week) |  |  |  |  |  |
| Deciduous forests |  |  |  |  | 0.295 |
| No | 50 (92.6) | 101 (96.2) | ref. |  |  |
| Yes | 4 (7.4) | 4 (3.8) | 2.26 | 0.49-10.42 |  |
|  |  |  |  |  |  |
| Coniferous forest |  |  |  |  | 0.351 |
| No | 51 (94.4) | 102 (97.1) | ref. |  |  |
| Yes | 3 (5.6) | 3 (2.9) | 2.38 | 0.38-14.97 |  |
|  |  |  |  |  |  |
| Mixed forests |  |  |  |  | **0.003** |
| No | 42 (77.8) | 98 (93.3) | ref. |  |  |
| Yes | 12 (22.2) | 7 (6.7) | 4.95 | 1.56-15.69 |  |
|  |  |  |  |  |  |
| Forest edges |  |  |  |  | **0.029** |
| No | 44 (81.5) | 96 (91.4) | ref. |  |  |
| Yes | 10 (18.5) | 9 (8.6) | 3.65 | 1.09-12.23 |  |
|  |  |  |  |  |  |
| Meadows/high grass |  |  |  |  | 0.384 |
| No | 47 (87.0) | 86 (81.9) | ref. |  |  |
| Yes | 7 (13.0) | 19 (18.1) | 0.58 | 0.17-1.99 |  |
|  |  |  |  |  |  |
| Town parks |  |  |  |  |  |
| No | 53 (98.2) | 96 (91.4) | ref. |  |  |
| Yes | 1 (1.8) | 9 (8.6) | - | - |  |
|  |  |  |  |  |  |
| City street |  |  |  |  | 0.483 |
| No | 47 (87.0) | 87 (82.9) | ref. |  |  |
| Yes | 7 (13.0) | 18 (17.1) | 0.67 | 0.21-2.10 |  |
|  |  |  |  |  |  |
| Cottage gardens |  |  |  |  | **0.005** |
| No | 52 (96.3) | 85 (81.0) | ref. |  |  |
| Yes | 2 (3.7) | 20 (19.0) | 0.18 | 0.04-0.78 |  |
|  |  |  |  |  |  |
| Fields / farms |  |  |  |  | 0.783 |
| No | 46 (85.2) | 89 (84.8) | ref. |  |  |
| Yes | 8 (14.8) | 16 (15.2) | 1.18 | 0.38-3.68 |  |
|  |  |  |  |  |  |
| Place of work time spent outdoors (>10 h/week) |  |  |  |  |  |
| Deciduous forests |  |  |  |  | 0.295 |
| No | 50 (92.6) | 97 (96.0) | ref. |  |  |
| Yes | 4 (7.4) | 4 (4.0) | 2.26 | 0.49-10.42 |  |
|  |  |  |  |  |  |
| Coniferous forest |  |  |  |  | 0.250 |
| No | 49 (90.7) | 96 (95.0) | ref. |  |  |
| Yes | 5 (9.3) | 5 (5.0) | 2.19 | 0.58-8.36 |  |
|  |  |  |  |  |  |
| Mixed forests |  |  |  |  | 0.401 |
| No | 50 (92.6) | 96 (95.0) | ref. |  |  |
| Yes | 4 (7.4) | 5 (5.0) | 2.00 | 0.40-9.91 |  |
|  |  |  |  |  |  |
| Forest edges |  |  |  |  | 0.686 |
| No | 51 (94.4) | 94 (93.1) | ref. |  |  |
| Yes | 3 (5.6) | 7 (6.9) | 0.72 | 0.14-3.74 |  |
|  |  |  |  |  |  |
| Meadows/high grass |  |  |  |  | - |
| No | 52 (96.3) | 91 (90.1) | - |  |  |
| Yes | 2 (3.7) | 10 (9.9) | - | - |  |
|  |  |  |  |  |  |
| Town parks |  |  |  |  | - |
| No | 54 (100) | 98 (97.0) | - |  |  |
| Yes | 0 (0) | 3 (3.0) | - | - |  |
|  |  |  |  |  |  |
| City streets |  |  |  |  | - |
| No | 54 (100) | 96 (95.0) | - |  |  |
| Yes | 0 (0) | 5 (5.0) | - | - |  |
|  |  |  |  |  |  |
| Cottage garden |  |  |  |  | - |
| No | 54 (100) | 99 (98.0) | - |  |  |
| Yes | 0 (0) | 2 (2.0) | - | - |  |
|  |  |  |  |  |  |
| Fields / farms |  |  |  |  | 0.447 |
| No | 50 (92.6) | 92 (91.1) | ref. |  |  |
| Yes | 4 (7.4) | 9 (8.9) | 0.60 | 0.15-2.34 |  |
|  |  |  |  |  |  |
| Place of total time spent outdoors (≥10 h/week) |  |  |  |  |  |
| Deciduous forests |  |  |  |  | 0.104 |
| No | 46 (88.5) | 98 (95.2) | ref. |  |  |
| Yes | 6 (11.5) | 5 (4.8) | 3.14 | 0.76-13.00 |  |
|  |  |  |  |  |  |
| Coniferous forest |  |  |  |  | 0.143 |
| No | 47 (90.4) | 99 (96.1) | ref. |  |  |
| Yes | 5 (9.6) | 4 (3.9) | 2.91 | 0.68-12.45 |  |
|  |  |  |  |  |  |
| Mixed forests |  |  |  |  | **0.008** |
| No | 40 (76.9) | 95 (92.2) | ref. |  |  |
| Yes | 12 (23.1) | 8 (7.8) | 3.97 | 1.37-11.54 |  |
|  |  |  |  |  |  |
| Forest edges |  |  |  |  | 0.253 |
| No | 42 (80.8) | 90 (87.4) | ref. |  |  |
| Yes | 10 (19.2) | 13 (12.6) | 1.78 | 0.66-4.75 |  |
|  |  |  |  |  |  |
| Meadows/high grass |  |  |  |  | 0.207 |
| No | 44 (84.6) | 79 (76.7) | ref. |  |  |
| Yes | 8 (15.4) | 24 (23.3) | 0.51 | 0.18-1.48 |  |
|  |  |  |  |  |  |
| Town parks |  |  |  |  | 0.152 |
| No | 46 (88.5) | 83 (80.6) | ref. |  |  |
| Yes | 6 (11.5) | 20 (19.4) | 0.44 | 0.13-1.43 |  |
|  |  |  |  |  |  |
| City street |  |  |  |  | **0.095** |
| No | 47 (90.4) | 84 (81.6) | ref. |  |  |
| Yes | 5 (9.6) | 19 (18.4) | 0.35 | 0.09-1.32 |  |
|  |  |  |  |  |  |
| Cottage garden |  |  |  |  | **0.007** |
| No | 50 (96.1) | 83 (80.6) | ref. |  |  |
| Yes | 2 (3.9) | 20 (19.4) | 0.18 | 0.04-0.82 |  |
|  |  |  |  |  |  |
| Fields / farms |  |  |  |  | 0.815 |
| No | 42 (80.8) | 83 (80.6) | ref. |  |  |
| Yes | 10 (19.2) | 20 (19.4) | 1.12 | 0.42-2.98 |  |
|  |  |  |  |  |  |
| Place of outdoor exposure during leisure time |  |  |  |  | **0.014** |
| <10h/week outdoors | 8 (14.8) | 13 (12.4) | ref. |  |  |
| >10h/week outdoors but <10h/week in forest | 34 (63.0) | 83 (79.0) | 0.72 | 0.25-2.04 |  |
| >10h/week in forest but <10h/week in mixed forest | 0 (0) | 2 (1.9) |  |  |  |
| >10h/week in mixed forest | 12 (22.2) | 7 (6.7) | 3.69 | 0.85-16.07 |  |
|  |  |  |  |  |  |
| Place of outdoor exposure in relation to work |  |  |  |  | 0.424 |
| <10h/week outdoors | 38 (70.4) | 81 (77.1) | ref. |  |  |
| >10h/week outdoors but <10h/week in forest | 10 (18.5) | 18 (17.1) | 1.54 | 0.56-4.21 |  |
| >10h/week in forest but <10h/week in mixed forest | 2 (3.7) | 1 (1.0) | 4.70 | 0.41-53.93 |  |
| >10h/week in mixed forest | 4 (7.4) | 5 (4.8) | 2.69 | 0.46-15.54 |  |
|  |  |  |  |  |  |
| Place of outdoor exposure in total |  |  |  |  | **0.040** |
| <10h/week outdoors | 5 (9.3) | 6 (5.7) | ref. |  |  |
| >10h/week outdoors but <10h/week in forest | 36 (66.7) | 89 (84.8) | 0.49 | 0.13-1.91 |  |
| >10h/week in forest but <10h/week in mixed forest | 1 (1.8) | 2 (1.9) | 0.92 | 0.05-15.69 |  |
| >10h/week in mixed forest | 12 (22.2) | 8 (7.6) | 2.16 | 0.42-11.17 |  |
|  |  |  |  |  |  |
| Consumption of unpasteurized cow milk or cheese |  |  |  |  | 0.211 |
| No | 42 (80.8) | 73 (70.9) | ref. |  |  |
| Yes | 10 (19.2) | 30 (29.1) | 0.60 | 0.26-1.36 |  |
|  |  |  |  |  |  |
| Consumption of unpasteurized sheep milk or cheese |  |  |  |  | - |
| No | 52 (100) | 102 (99.0) | - |  |  |
| Yes | 0 (0) | 1 (1.0) | - | - |  |
|  |  |  |  |  |  |
| Consumption of unpasteurized goat milk or cheese |  |  |  |  | - |
| No | 52 (100) | 101 (98.1) | - | - |  |
| Yes | 0 (0) | 2 (1.9) | - | - |  |
|  |  |  |  |  |  |
| Exposure to animals and tick bites |  |  |  |  |  |
| Contact with dog |  |  |  |  | - |
| No | 4 (9.8) | 6 (6.8) | - | - |  |
| Yes | 37 (90.2) | 82 (93.2) | - | - |  |
|  |  |  |  |  |  |
| Contact with cat |  |  |  |  | **0.026** |
| No | 24 (61.5) | 37 (42.0) | ref. |  |  |
| Yes | 15 (38.5) | 51 (58.0) | 0.40 | 0.17-0.92 |  |
|  |  |  |  |  |  |
| Found ticks on domestic animal |  |  |  |  | 0.422 |
| No | 23 (56.1) | 52 (60.5) | ref. |  |  |
| Yes | 18 (43.9) | 34 (39.5) | 1.40 | 0.61-3.20 |  |
|  |  |  |  |  |  |
| Reported exposure to tick bite |  |  |  |  | **<0.001** |
| No | 17 (32.7) | 92 (89.3) | ref. |  |  |
| Yes | 35 (67.3) | 11 (10.7) | 26.82 | 6.39-112.58 |  |
|  |  |  |  |  |  |
| Known place of exposure to ticks: |  |  |  |  | **<0.001** |
| No exposure to ticks | 17 (32.7) | 92 (89.3) | ref. |  |  |
| Near residence | 33 (63.5) | 10 (9.7) | 51.54 | 7.00-379.72 |  |
| During travel to endemic region | 2 (3.8) | 0 (0) | - | - |  |
| During travel to non endemic region | 0 (0) | 1 (1.0) | - | - |  |

* column percentages calculated excluding missing observations; † there were only forestry workers in the studied population
