# Supplementary material for: A National Case-Control Study Identifies Human Socio-Economic Status and Activities as Risk Factors for Tick-Borne Encephalitis in Poland
Source: PLoS One. 2012 Sep 19;7(9):e45511. doi: 10.1371/journal.pone.0045511 (PMC3446880; doi:10.1371/journal.pone.0045511)
Supplement: Table S4 — Selection of the form of ordered variables (endemic areas). (DOCX) [file pone.0045511.s006.docx]

**Table S4. Selection of the form of ordered variables (endemic areas)**

Univariate analysis of ordinal variables in endemic areas was performed to select the best variable form. The variable form was also checked in the intermediate and candidate final models, giving similar results (data not shown). In majority of cases the variable form with the smallest AIC was considered (shaded), even though in some cases the differences in the information criteria values were not sufficient to discriminate between the models. In case AIC pointed to score variable but was very near to the value of the dichotomous variable, the later form was used for simplicity of interpretation (forest proximity, leisure time outdoors).

| **Variable** | **Category description** | **AIC** | **BIC** | **Univariate p-value** |
| --- | --- | --- | --- | --- |
| **Education** | categorical, 4 categories | 248.86 | 264.23 | 0.070 |
|  | categorical, 2 categories (secondary or higher; primary or vocational) | 245.56 | 249.40 | 0.015 |
|  | categorical, 2 categories (higher; primary/ secondary/ vocational) | 247.25 | 251.09 | 0.039 |
|  | Score | 250.69 | 254.53 | 0.365 |
|  |  |  |  |  |
| **Income** | categorical, 4 categories | 254.53 | 266.07 | 0.456 |
|  | categorical, 2 categories (≤480; >480 USD) | 250.62 | 254.47 | 0.113 |
|  | Score | 251.57 | 255.42 | 0.211 |
|  |  |  |  |  |
| **Forest proximity** | categorical, 5 categories | 247.08 | 262.43 | 0.043 |
|  | categorical, 2 categories (≤500m; >500m) | 247.58 | 251.42 | 0.067 |
|  | categorical, 2 categories (≤1km; >1km) | 244.77 | 248.60 | 0.013 |
|  | Score | 243.64 | 247.47 | 0.007 |
|  |  |  |  |  |
| **Leisure time spent outdoors** | categorical, 6 categories | 254.88 | 270.26 | 0.372 |
|  | categorical, 2 categories (<20h; ≥20h) | 251.58 | 255.43 | 0.212 |
|  | Score | 251.03 | 254.87 | 0.146 |
|  |  |  |  |  |
| **Work time spent outdoors** | categorical, 6 categories | 255.95 | 275.18 | 0.393 |
|  | categorical, 2 categories (<20h; ≥20h) | 250.80 | 254.65 | 0.126 |
|  | score | 251.08 | 254.93 | 0.152 |
|  |  |  |  |  |
| **Time spent outdoors** | categorical, 4 categories | 255.33 | 266.86 | 0.803 |
|  | categorical, 2 categories (<40h; ≥40h) | 251.38 | 255.22 | 0.331 |
|  | score | 251.78 | 255.63 | 0.462 |
|  |  |  |  |  |
| **Time of travel** | categorical, 4 categories (no travel; 1-5d; 5-15d; >15d) | 245.37 | 256.86 | 0.068 |
|  | categorical, 3 categories (no travel, 1-5d; >5d) | 245.27 | 252.93 | 0.073 |
|  | categorical, 2 categories (no travel or <5d; >5d) | 246.22 | 250.07 | 0.009 |
|  | numerical (number of days) | 247.61 | 251.44 | 0.345 |
|  |  |  |  |  |
| **Time travelling to endemic areas** | categorical, 3 categories (no travel, 1-5d; >5d) | 251.20 | 258.87 | 0.706 |
|  | categorical, 2 categories (no travel; any travel) | 250.07 | 253.90 | 0.350 |
|  | numerical (number of days) | 249.89 | 253.73 | 0.967 |
|  |  |  |  |  |
| **Time travelling to non-endemic areas** | categorical, 3 categories (no travel, 1-5d; >5d) | 247.03 | 254.71 | 0.052 |
|  | categorical, 2 categories (no travel; any travel) | 246.42 | 250.26 | 0.034 |
|  | numerical (number of days) | 250.22 | 254.06 | 0.396 |

h – numer of hours; d- number of days
