# Supplementary material for: A National Case-Control Study Identifies Human Socio-Economic Status and Activities as Risk Factors for Tick-Borne Encephalitis in Poland
Source: PLoS One. 2012 Sep 19;7(9):e45511. doi: 10.1371/journal.pone.0045511 (PMC3446880; doi:10.1371/journal.pone.0045511)
Supplement: Table S5 — Effect of main socio-economic factors in endemic regions. (DOCX) [file pone.0045511.s007.docx]

**Table S5. Effect of main socio-economic factors in endemic regions.**

Below we summarize an intermediate model containing only socio-economic factors. Due to limited sample size it was not possible to keep all the categories for occupation. Occupations with similar odds ratios were grouped, apart from foresters, who were kept separate as they are typically considered a high-risk occupation. Odds ratios giving p-values <0.10 are shown in bold.

| **Variable** | **Coding** | **Odds Ratio** | **S.E.** | **Z** | **p-value** | **95% Confidence Interval** |
| --- | --- | --- | --- | --- | --- | --- |
| **Adult** | Yes/No | 0.96 | 1.42 | -0.03 | 0.977 | 0.05-17.43 |
|  |  |  |  |  |  |  |
| **Education (among adults)** | high school or higher vs. primary /vocational | **0.61** | **0.17** | **-1.77** | **0.077** | **0.35-1.06** |
|  |  |  |  |  |  |  |
| **Income per household member (USD)** | >480 vs. ≤480 | 0.78 | 0.43 | -0.45 | 0.650 | 0.26-2.30 |
|  |  |  |  |  |  |  |
| **Occupation (among adults)** | Students | Ref. |  |  |  |  |
|  | Managers | 0.00 | 0.00 | -0.02 | 0.984 | - |
|  | Professionals | 1.30 | 1.75 | 0.19 | 0.849 | 0.09-18.41 |
|  | Technicians and associate professionals | 4.66 | 5.05 | 1.42 | 0.155 | 0.56-38.94 |
|  | Clerical support workers | 0.99 | 1.19 | -0.01 | 0.994 | 0.09-10.43 |
|  | Service and sales workers | 2.48 | 2.90 | 0.78 | 0.438 | 0.25-24.51 |
|  | Agricultural workers | 1.26 | 1.28 | 0.23 | 0.822 | 0.17-9.24 |
|  | Forestry or fishery workers | 5.78 | 6.65 | 1.53 | 0.127 | 0.61-55.08 |
|  | Craft and related trades workers | 3.78 | 3.89 | 1.29 | 0.197 | 0.50-28.44 |
|  | Plant and machine operators, and assemblers | 2.61 | 2.77 | 0.90 | 0.368 | 0.32-20.93 |
|  | Elementary occupations | 4.40 | 4.68 | 1.39 | 0.163 | 0.55-35.35 |
|  | Unemployed | **7.08** | **7.61** | **1.82** | **0.069** | **0.86-58.24** |
|  | Retired | 1.55 | 1.75 | 0.39 | 0.697 | 0.17-14.15 |
