# Supplementary material for: A National Case-Control Study Identifies Human Socio-Economic Status and Activities as Risk Factors for Tick-Borne Encephalitis in Poland
Source: PLoS One. 2012 Sep 19;7(9):e45511. doi: 10.1371/journal.pone.0045511 (PMC3446880; doi:10.1371/journal.pone.0045511)
Supplement: Table S6 — Main socio-economic factors in endemic regions. (DOCX) [file pone.0045511.s008.docx]

**Table S6. Main socio-economic factors in endemic regions**.

Results of grouping certain occupation variables.

| **Variable** | **Coding** | **Odds Ratio** | **S.E.** | **Z** | **p-value** | **95% Confidence Interval** |
| --- | --- | --- | --- | --- | --- | --- |
| **Adult** | Yes/No | 1.15 | 1.59 | 0.10 | 0.922 | 0.08-17.44 |
|  |  |  |  |  |  |  |
| **Education (among adults)** | High school or higher vs primary /vocational | **0.62** | **0.17** | **-1.76** | **0.079** | **0.36-1.06** |
|  |  |  |  |  |  |  |
| **Income per household member (USD)** | >480 vs ≤480 | 0.70 | 0.36 | -0.70 | 0.486 | 0.26-1.91 |
|  |  |  |  |  |  |  |
| **Occupation** | Technicians, craftsmen and elementary occupations | **2.81** | **0.99** | **2.94** | **0.003** | **1.41-5.59** |
|  | Forestry or fishery workers* | **3.96** | **2.60** | **2.10** | **0.036** | **1.09-14.34** |
|  | Unemployed | **4.77** | **2.47** | **3.01** | **0.003** | **1.73-13.15** |
|  | Other status (including students and retired) | ref. |  |  |  |  |

* there were only forestry workers in the studied population
