# Supplementary material for: A National Case-Control Study Identifies Human Socio-Economic Status and Activities as Risk Factors for Tick-Borne Encephalitis in Poland
Source: PLoS One. 2012 Sep 19;7(9):e45511. doi: 10.1371/journal.pone.0045511 (PMC3446880; doi:10.1371/journal.pone.0045511)
Supplement: Table S7 — Effect of place of residence and travel history in endemic regions. (DOCX) [file pone.0045511.s009.docx]

**Table S7. Effect of place of residence and travel history in endemic regions.**

An intermediate model with residence place characteristics and travel history. Although in the further analysis travel to non-endemic regions was considered as an indicator variable, due to information criteria and small number of respondents who traveled during the exposure period, it is worth noting that this variable displays a dose effect with larger protective effect of longer journeys.

| **Variable** | **Coding** | **Odds Ratio** | **S.E.** | **Z** | **p-value** | **95% Confidence Interval** |
| --- | --- | --- | --- | --- | --- | --- |
| **Distance from residence to nearest forest** | >500 m vs. ≤500 m | **0.63** | **0.15** | **-1.94** | **0.052** | **0.39-1.00** |
| **Living on a farm** | Yes/No | 0.78 | 0.21 | -0.90 | 0.368 | 0.46-1.33 |
| **Living in a house with yard or garden** | Yes/No | 0.68 | 0.26 | -1.02 | 0.309 | 0.33-1.43 |
| **Travel to an endemic area during exposure period** | Yes/No | 0.80 | 0.26 | -0.70 | 0.483 | 0.42-1.50 |
| **Travel to non-endemic area during exposure period** | Yes, travel duration <5days | 0.63 | 0.26 | -1.13 | 0.258 | 0.28-1.40 |
|  | Yes, travel duration ≥5days | **0.33** | **0.19** | **-1.87** | **0.061** | **0.10-1.05** |
|  | No | ref. |  |  |  |  |
