# Supplementary material for: A National Case-Control Study Identifies Human Socio-Economic Status and Activities as Risk Factors for Tick-Borne Encephalitis in Poland
Source: PLoS One. 2012 Sep 19;7(9):e45511. doi: 10.1371/journal.pone.0045511 (PMC3446880; doi:10.1371/journal.pone.0045511)
Supplement: Table S8 — Independent effects of total time spent at different locations and (for selected variables) in relation to work or recreation during exposure period in endemic regions. (DOCX) [file pone.0045511.s010.docx]

**Table S8. Independent effects of time spent at different locations in total and (for selected variables) in relation to work or recreation during exposure period in endemic regions**

Stepwise selection procedure (p<0.1 for retention) from this intermediate model (model A) leaves only the effects of mixed forest and forest edge. Therefore in model B we considered both recreational and occupational exposure in these locations. Forest edge exposure during leisure time was dropped at this time.

| **Model** | **Variable** | **Odds Ratio** | **S.E** | **Z** | **p-value** | **95% Confidence Interval** |
| --- | --- | --- | --- | --- | --- | --- |
| **Model A: Time spent outdoors in total (≥10 h/week)** | deciduous forest | 0.99 | 0.76 | -0.01 | 0.991 | 0.22-4.50 |
|  | coniferous forest | 0.43 | 0.31 | -1.16 | 0.247 | 0.10-1.81 |
|  | mixed forest | **3.99** | **1.65** | **3.34** | **0.001** | **1.77-8.97** |
|  | forest edge | 0.59 | 0.28 | -1.11 | 0.269 | 0.23-1.51 |
|  | meadows/high grass | 0.68 | 0.29 | -0.89 | 0.372 | 0.30-1.58 |
|  | town parks | 0.58 | 0.43 | -0.74 | 0.459 | 0.13-2.47 |
|  | city streets | 0.64 | 0.43 | -0.67 | 0.502 | 0.17-2.36 |
|  | cottage garden | 0.82 | 0.36 | -0.45 | 0.656 | 0.34-1.96 |
|  | fields/ farms | 0.54 | 0.24 | -1.39 | 0.164 | 0.23-1.29 |
|  |  |  |  |  |  |  |
| **Model B: Time spent outdoors in relation to work (≥10 h/week)** | mixed forest | **3.45** | **2.32** | **1.84** | **0.066** | **0.92-12.92** |
|  | forest edge | **0.21** | **0.15** | **-2.20** | **0.028** | **0.05-0.85** |
|  |  |  |  |  |  |  |
| **Model B: Time spent outdoors during leisure activities (≥10 h/week)** | mixed forest | **3.31** | **1.56** | **2.54** | **0.011** | **1.32-8.34** |
|  | forest edge | 0.77 | 0.46 | -0.44 | 0.656 | 0.24-2.47 |
