# Supplementary material for: A National Case-Control Study Identifies Human Socio-Economic Status and Activities as Risk Factors for Tick-Borne Encephalitis in Poland
Source: PLoS One. 2012 Sep 19;7(9):e45511. doi: 10.1371/journal.pone.0045511 (PMC3446880; doi:10.1371/journal.pone.0045511)
Supplement: Table S9 — Independent effects of spending leisure time in particular outdoor activities during exposure period in endemic region. (DOCX) [file pone.0045511.s011.docx]

**Table S9. Independent effects of spending leisure time in particular outdoor activities during exposure period in endemic region.**

Despite presenting independent effects of different activities outdoors, we note that certain effects were highly correlated – in particular sailing and camping - which may explain the strong effect of the former.

| **Model** | **Variable** | **Odds Ratio** | **S.E** | **Z** | **p-value** | **95% Conf. Interval** |
| --- | --- | --- | --- | --- | --- | --- |
| **Model A** | Hunting | 1.46 | 1.09 | 0.51 | 0.613 | 0.34-6.28 |
|  | Camping | **0.14** | **0.09** | **-3.02** | **0.003** | **0.04-0.50** |
|  | Fishing | 1.04 | 0.36 | 0.11 | 0.915 | 0.53-2.04 |
|  | Swimming outdoors | **0.42** | **0.17** | **-2.20** | **0.028** | **0.19-0.91** |
|  | Sailing | 7.28 | 6.13 | 2.36 | 0.018 | 1.40-37.92 |
|  | Hiking | 1.01 | 0.26 | 0.05 | 0.957 | 0.62-1.66 |
|  | Cycling | 1.02 | 0.29 | 0.08 | 0.936 | 0.58-1.79 |
|  | Collecting mushrooms or berries | **2.00** | **0.56** | **2.46** | **0.014** | **1.15-3.48** |
|  | Gardening | 0.91 | 0.26 | -0.32 | 0.750 | 0.52-1.59 |
|  |  |  |  |  |  |  |
| **Model B (after stepwise procedure)** | Sailing | **7.44** | **6.18** | **2.42** | **0.016** | **1.46-37.86** |
|  | Camping | **0.14** | **0.09** | **-3.06** | **0.002** | **0.04-0.49** |
|  | Collecting mushrooms or berries | **2.00** | **0.55** | **2.53** | **0.011** | **1.17-3.42** |
|  | Swimming outdoors | **0.44** | **0.17** | **-2.18** | **0.029** | **0.21-0.92** |

Based on the univariate analysis and the above intermediate models (Tables S5-S9) the following candidate variables were considered for the final model: sailing (yes/no), camping (yes/no), collecting mushrooms/berries (yes/no), swimming outdoors (yes/no),<10h/week spent in mixed forest in relation to work (yes/no), >10h/week spent in mixed forest during leisure activities (yes/no), >10h/week spent at forest edge in relation to work (yes/no), distance from residence to forest (<=500 m, >500 m), travel to non-endemic area (yes/no), occupation (technicians, craftsmen and elementary occupations; forestry or fishery workers; unemployed; others), education (high school or higher; primary /vocational).
