# Supplementary material for: A National Case-Control Study Identifies Human Socio-Economic Status and Activities as Risk Factors for Tick-Borne Encephalitis in Poland
Source: PLoS One. 2012 Sep 19;7(9):e45511. doi: 10.1371/journal.pone.0045511 (PMC3446880; doi:10.1371/journal.pone.0045511)
Supplement: Table S10 — Model containing main effects for the candidate variables – endemic area. (DOCX) [file pone.0045511.s012.docx]

**Table S10. Model containing main effects for the candidate variables – endemic area**

| **Variable** | **Coding** | **OR** | **95% CI** | **p-value** |
| --- | --- | --- | --- | --- |
| **Education** | secondary/higher | 0.66 | 0.35-1.25 | 0.206 |
|  | vocational/primary | Ref. |  |  |
|  |  |  |  |  |
| **Occupation** | Technicians, craftsmen and elementary occupations | **5.53** | **2.10-14.55** | **0.001** |
|  | Forestry or fishery workers | **7.74** | **0.92-64.82** | **0.059** |
|  | Unemployed | **11.70** | **2.79-49.08** | **0.001** |
|  | Other | ref. |  |  |
|  |  |  |  |  |
| **Residence distance from forest** | >500 m vs. ≤500 m | **0.46** | **0.25-0.84** | **0.012** |
|  |  |  |  |  |
| **Travel to non-endemic areas** | yes vs no | **0.36** | **0.14-0.90** | **0.031** |
|  |  |  |  |  |
| **≥10h/week at forest edge in relation to work** | yes vs no | **0.14** | **0.03-0.63** | **0.010** |
|  |  |  |  |  |
| **≥10h/week in mixed forest in relation to work** | yes vs no | 1.00 | 0.16-6.22 | 0.998 |
|  |  |  |  |  |
| **≥10h/week in mixed forest during leisure time** | yes vs no | **4.37** | **1.45-13.12** | **0.009** |
|  |  |  |  |  |
| **Collecting forest foods** | yes vs no | 1.52 | 0.77-2.97 | 0.225 |
|  |  |  |  |  |
| **Camping** | yes vs no | **0.12** | **0.03-0.54** | **0.006** |
|  |  |  |  |  |
| **Swimming** | yes vs no | **0.26** | **0.10-0.66** | **0.004** |
|  |  |  |  |  |
| **Sailing** | yes vs no | **5.80** | **0.88-38.30** | **0.068** |

Backwards selection procedure with p=0.05 as the cut off was used and the interactions between specific activities and the places visited during leisure time were examined to obtain the final model.
