# Supplementary material for: A National Case-Control Study Identifies Human Socio-Economic Status and Activities as Risk Factors for Tick-Borne Encephalitis in Poland
Source: PLoS One. 2012 Sep 19;7(9):e45511. doi: 10.1371/journal.pone.0045511 (PMC3446880; doi:10.1371/journal.pone.0045511)
Supplement: Table S11 — Selection of the form of ordered variables (non-endemic areas). (DOCX) [file pone.0045511.s013.docx]

**Table S11. Selection of the form of ordered variables (non-endemic areas)**

Univariate analysis for ordinal variables in non-endemic areas in order was performed to select the best variable form. The variable form was also checked in the intermediate and candidate final models and on one occasion a different variable form was selected (residence distance from forests) (data not shown). The variable form with the smallest AIC was considered (shaded), even though in some cases the differences in the information criteria values were not sufficient to discriminate between the models.

| **Variable** | **Category description** | **AIC** | **BIC** | **Univariate p-value** |
| --- | --- | --- | --- | --- |
| **Education** | categorical, 4 categories | 114.38 | 126.50 | 0.329 |
|  | categorical, 2 categories (secondary or higher; elementary or vocational) | 111.98 | 115.01 | 0.315 |
|  | categorical, 2 categories (higher; elementary, secondary or vocational) | 112.01 | 115.04 | 0.323 |
|  | Score | 109.32 | 112.35 | 0.056 |
|  |  |  |  |  |
| **Income** | categorical, 4 categories | 119.97 | 129.18 | 0.703 |
|  | categorical, 2 categories (≤480; >480 USD) | 116.46 | 119.53 | 0.338 |
|  | Score | 116.71 | 119.78 | 0.412 |
|  |  |  |  |  |
| **Forest proximity** | categorical, 5 categories | 119.13 | 131.40 | 0.373 |
|  | categorical, 2 categories (≤500m; >500m) | 113.32 | 116.38 | 0.044 |
|  | categorical, 2 categories (≤1km; >1km) | 116.29 | 119.36 | 0.296 |
|  | Score | 114.48 | 117.55 | 0.089 |
|  |  |  |  |  |
| **Leisure time spent outdoors** | categorical, 6 categories | 117.61 | 129.89 | 0.217 |
|  | categorical, 2 categories (<20h; ≥20h) | 115.30 | 118.37 | 0.149 |
|  | Score | 117.31 | 120.38 | 0.794 |
|  |  |  |  |  |
| **Work time spent outdoors** | categorical, 6 categories | 118.72 | 134.06 | 0.247 |
|  | categorical, 2 categories (<20h; ≥20h) | 117.28 | 120.35 | 0.751 |
|  | Score | 117.28 | 120.35 | 0.749 |
|  |  |  |  |  |
| **Time spent outdoors** | categorical, 4 categories | 115.76 | 124.85 | 0.747 |
|  | categorical, 2 categories (<40h; ≥40h) | 112.68 | 115.71 | 0.577 |
|  | Score | 112.98 | 116.01 | 0.921 |
|  |  |  |  |  |
| **Time of travel** | categorical, 4 categories (no travel; 1-5d; 5-15d,>15d) | 119.06 | 128.25 | 0.680 |
|  | categorical, 3 categories (no travel; 1-5d; >5d) | 117.10 | 123.22 | 0.479 |
|  | categorical, 2 categories (no travel or <5d; >5d) | 116.81 | 119.88 | 0.449 |
|  | numerical (number of days) | 114.74 | 117.81 | 0.177 |
|  |  |  |  |  |
| **Time travelling to endemic areas** | categorical, 3 categories (no travel; 1-5d; >5d) | 117.48 | 123.60 | 0.578 |
|  | categorical, 2 categories (no travel; any travel) | 113.42 | 116.48 | 0.126 |
|  | numerical (number of days) | 115.41 | 118.47 | 0.281 |
|  |  |  |  |  |
| **Time travelling to non-endemic areas** | categorical, 3 categories (no travel; 1-5d; >5d) | 113.99 | 120.12 | 0.101 |
|  | categorical, 2 categories (no travel; any travel) | 111.40 | 114.46 | 0.037 |
|  | numerical (number of days) | 113.74 | 116.80 | 0.093 |

h – number of hours; d – number of days
