# Supplementary material for: A National Case-Control Study Identifies Human Socio-Economic Status and Activities as Risk Factors for Tick-Borne Encephalitis in Poland
Source: PLoS One. 2012 Sep 19;7(9):e45511. doi: 10.1371/journal.pone.0045511 (PMC3446880; doi:10.1371/journal.pone.0045511)
Supplement: Table S12 — Effect of main socio-economic factors in non-endemic regions. (DOCX) [file pone.0045511.s014.docx]

**Table S12. Effect of main socio-economic factors in non-endemic regions.**

| **Variable** | **Coding** | **Odds Ratio** | **S.E.** | **Z** | **p-value** | **95% Confidence Interval** |
| --- | --- | --- | --- | --- | --- | --- |
| **Adult** | Yes/No | 0.81 | 2.04 | -0.08 | 0.933 | 0.01-112.15 |
|  |  |  |  |  |  |  |
| **Education (among adults)** | Score (per category increase) | 0.66 | 0.18 | -1.49 | 0.135 | 0.38-1.14 |
|  |  |  |  |  |  |  |
| **Income per household member (USD)** | >480 vs ≤480 | 0.71 | 0.57 | -0.43 | 0.667 | 0.14-3.45 |
|  |  |  |  |  |  |  |
| **Occupation (among adults)** | Students | 0.94 | 1.90 | -0.03 | 0.975 | 0.02-50.08 |
|  | Managers | 0.00 | 0.00 | 0.00 | 0.996 |  |
|  | Professionals | 0.64 | 1.13 | -0.25 | 0.799 | 0.02-21.00 |
|  | Technicians and associate professionals | 0.68 | 1.14 | -0.23 | 0.819 | 0.03-18.01 |
|  | Clerical support workers | NE | - | 0.01 | 0.992 | - |
|  | Service and sales workers | 15.97 | 35.81 | 1.24 | 0.216 | 0.2-1292.25 |
|  | Agricultural workers | 3.25 | 3.86 | 0.99 | 0.321 | 0.32-33.34 |
|  | Forestry or fishery workers* | **23.46** | **44.85** | **1.65** | **0.099** | **0.55-995.15** |
|  | Craft and related trades workers | 2.66 | 4.40 | 0.59 | 0.553 | 0.1-67.92 |
|  | Plant and machine operators, and assemblers | 6.20 | 10.39 | 1.09 | 0.276 | 0.23-165.21 |
|  | Elementary occupations | NE | - | 0.01 | 0.992 | - |
|  | Unemployed | 0.72 | 1.21 | -0.20 | 0.843 | 0.03-19.32 |
|  | Retired | ref. |  |  |  |  |

NE – not estimable due to extremely low numbers; * there were only forestry workers in the studied population
