# Supplementary material for: A National Case-Control Study Identifies Human Socio-Economic Status and Activities as Risk Factors for Tick-Borne Encephalitis in Poland
Source: PLoS One. 2012 Sep 19;7(9):e45511. doi: 10.1371/journal.pone.0045511 (PMC3446880; doi:10.1371/journal.pone.0045511)
Supplement: Table S13 — Main socio-economic factors in non-endemic regions – proposed grouping of certain occupation variables. (DOCX) [file pone.0045511.s015.docx]

**Table S13. Main socio-economic factors in non-endemic regions – proposed grouping of certain occupation variables.**

Due to limited sample size we were not able to study potentially important effects of some of the occupational groups. We decided to keep only the effect of working as a forest worker (below).

| **Variable** | **Coding** | **Odds Ratio** | **S.E.** | **Z** | **p-value** | **95% Confidence Interval** |
| --- | --- | --- | --- | --- | --- | --- |
| **Adult** | Yes/No | 0.79 | 1.12 | -0.17 | 0.866 | 0.05-13.01 |
|  |  |  |  |  |  |  |
| **Education (among adults)** | Score (per category increase) | **0.64** | **0.14** | **-1.99** | **0.046** | **0.41-0.99** |
|  |  |  |  |  |  |  |
| **Income per household member (USD)** | >480 vs ≤480 | 1.14 | 0.77 | 0.20 | 0.844 | 0.31-4.25 |
|  |  |  |  |  |  |  |
| **Occupation** | Forestry or fishery workers vs other status | 6.61 | 8.03 | 1.55 | 0.120 | 0.61-71.57 |
