# Supplementary material for: A National Case-Control Study Identifies Human Socio-Economic Status and Activities as Risk Factors for Tick-Borne Encephalitis in Poland
Source: PLoS One. 2012 Sep 19;7(9):e45511. doi: 10.1371/journal.pone.0045511 (PMC3446880; doi:10.1371/journal.pone.0045511)
Supplement: Table S14 — Effect of place of residence and travel history in non-endemic regions. (DOCX) [file pone.0045511.s016.docx]

**Table S14. Effect of place of residence and travel history in non-endemic regions.**

Distance of residence from forest and travel might be important factors. We note a non- significant dose effect for travelling to non-endemic areas. Travel to endemic areas was not significant despite considerable effect size due to small number of participants who traveled to endemic areas. However, due to possible confounding, this effect was kept in the models.

| **Variable** | **Coding** | **Odds Ratio** | **S.E.** | **Z** | **p-value** | **95% Confidence Interval** |
| --- | --- | --- | --- | --- | --- | --- |
| **Distance from residence to nearest forest** | >500 m vs ≤500 m | **2.79** | **1.31** | **2.19** | **0.029** | 1.11-7.02 |
|  |  |  |  |  |  |  |
| **Living on a farm** | Yes/No | 1.61 | 0.76 | 1.02 | 0.308 | 0.64-4.05 |
|  |  |  |  |  |  |  |
| **Living in a house with yard or garden** | Yes/No | 0.57 | 0.31 | -1.04 | 0.299 | 0.20-1.65 |
|  |  |  |  |  |  |  |
| **Travel to an endemic area during exposure period** | Yes/No | 3.23 | 3.06 | 1.24 | 0.215 | 0.51-20.66 |
|  |  |  |  |  |  |  |
| **Travel to non-endemic area during exposure period** | Yes, travel duration <5 days | 0.56 | 0.35 | -0.93 | 0.350 | 0.17-1.88 |
|  | Yes, travel duration ≥5 days | 0.35 | 0.29 | -1.25 | 0.211 | 0.07-1.81 |
|  | No | ref. |  |  |  |  |
