# Supplementary material for: A National Case-Control Study Identifies Human Socio-Economic Status and Activities as Risk Factors for Tick-Borne Encephalitis in Poland
Source: PLoS One. 2012 Sep 19;7(9):e45511. doi: 10.1371/journal.pone.0045511 (PMC3446880; doi:10.1371/journal.pone.0045511)
Supplement: Table S15 — Independent effects of total time spent at different locations and (for selected variables) in relation to work or recreation during exposure period in non-endemic regions. (DOCX) [file pone.0045511.s017.docx]

**Table S15. Independent effects of time spent at different locations in total and (for selected variables) in relation to work or recreation during exposure period in non-endemic regions**

Backwards selection procedure from model A (p for removal 0.05) leaves time spent in mixed forest (OR 4.08; 95% CI 1.36 – 12.26) and time spent in cottage gardens (OR 0.18; 0.04 – 0.81) (not shown in the table). These locations were considered in model B with break up for leisure and work activities. Occupational exposure in mixed forest had less effect. Spending time in gardens was protective for both, but rarely done in relation to work.

| **Variable** | **Coding** | **Odds Ratio** | **S.E.** | **Z** | **p-value** | **95% Confidence Interval** |
| --- | --- | --- | --- | --- | --- | --- |
| **Model A: Time spent outdoors in total (≥10 h/week)** | deciduous forest | 0.45 | 0.77 | -0.47 | 0.642 | 0.02-12.68 |
|  | coniferous forest | 4.40 | 8.05 | 0.81 | 0.418 | 0.12-158.75 |
|  | mixed forest | 3.54 | 2.83 | 1.58 | 0.114 | 0.74-17.00 |
|  | forest edge | 1.64 | 1.25 | 0.64 | 0.521 | 0.36-7.33 |
|  | meadows/high grass | 0.29 | 0.22 | -1.62 | 0.105 | 0.07-1.29 |
|  | town parks | 0.82 | 0.88 | -0.18 | 0.855 | 0.10-6.67 |
|  | city streets | 0.25 | 0.33 | -1.04 | 0.297 | 0.02-3.37 |
|  | cottage garden | **0.20** | **0.16** | **-2.05** | **0.041** | **0.04-0.94** |
|  | fields/farms | 1.00 | 0.67 | -0.01 | 0.995 | 0.27-3.74 |
|  |  |  |  |  |  |  |
|  |  |  |  |  |  |  |
| **Model B-1: Time spent outdoors in relation to work (≥10 h/week)** | mixed forest | 1.23 | 1.28 | 0.20 | 0.842 | 0.16-9.51 |
|  | Cottage gardens | 0.00 | 0.00 | -0.01 | 0.993 |  |
|  |  |  |  |  |  |  |
| **Model B-2: Time spent outdoors during leisure activities (≥10 h/week)** | mixed forest | **4.78** | **3.11** | **2.40** | **0.016** | **1.33-17.11** |
|  | Cottage gardens | **0.18** | **0.14** | **-2.24** | **0.025** | **0.04-0.80** |
