# Supplementary material for: A National Case-Control Study Identifies Human Socio-Economic Status and Activities as Risk Factors for Tick-Borne Encephalitis in Poland
Source: PLoS One. 2012 Sep 19;7(9):e45511. doi: 10.1371/journal.pone.0045511 (PMC3446880; doi:10.1371/journal.pone.0045511)
Supplement: Table S16 — Independent effects of spending leisure time on particular outdoor activities during exposure period in non-endemic region. (DOCX) [file pone.0045511.s018.docx]

**Table S16. Independent effects of spending leisure time on particular outdoor activities during exposure period in non-endemic region.**

No particular recreational activity was identified as a risk factor. Backwards selection procedure did not leave any effect at the significance level of p<0.1

|  | **Variable** | **Odds Ratio** | **S.E.** | **Z** | **p-value** | **95% Confidence Interval** |
| --- | --- | --- | --- | --- | --- | --- |
| **Recreational activity** | Hunting | 3.30 | 4.35 | 0.91 | 0.364 | 0.25-43.64 |
|  | Camping | 4.63 | 4.58 | 1.55 | 0.121 | 0.67-32.17 |
|  | Fishing | 1.34 | 0.85 | 0.47 | 0.641 | 0.39-4.66 |
|  | Swimming outdoors | 0.58 | 0.35 | -0.90 | 0.366 | 0.18-1.89 |
|  | Sailing | 0.23 | 0.32 | -1.06 | 0.291 | 0.02-3.50 |
|  | Hiking | 0.56 | 0.23 | -1.40 | 0.163 | 0.25-1.26 |
|  | Cycling | 0.92 | 0.37 | -0.20 | 0.845 | 0.42-2.02 |
|  | Collecting forest foods | 1.15 | 0.46 | 0.35 | 0.724 | 0.53-2.53 |
|  | Gardening | 0.73 | 0.31 | -0.75 | 0.455 | 0.31-1.68 |

Based on the univariate analysis and the intermediate models (Tables S12-S16), the following candidate variables were included in the initial full multivariate model: education (per one level increase), occupation (forester vs other), residence distance from the forest (≤500 m vs >500 m), travel to non-endemic area (yes/no), travel to endemic area (yes/no), ≥10h/week in mixed forest during leisure time (yes/no), ≥10h/week in cottage gardens (yes/no) (Table S17).
