# Supplementary material for: A National Case-Control Study Identifies Human Socio-Economic Status and Activities as Risk Factors for Tick-Borne Encephalitis in Poland
Source: PLoS One. 2012 Sep 19;7(9):e45511. doi: 10.1371/journal.pone.0045511 (PMC3446880; doi:10.1371/journal.pone.0045511)
Supplement: Table S17 — Model containing main effects for the candidate variables in non-endemic areas. (DOCX) [file pone.0045511.s019.docx]

**Table S17. Model containing main effects for the candidate variables in non-endemic areas**

|  |  | **Odds Ratio** | **S.E.** | **Z** | **p-value** | **95% Confidence Interval** |
| --- | --- | --- | --- | --- | --- | --- |
| **Education** | (per one level increase) | 0.68 | 0.18 | -1.48 | 0.139 | 0.41-1.13 |
| **Occupation** | Forester vs other | 3.31 | 4.89 | 0.81 | 0.418 | 0.18-59.99 |
| **Residence distance from forest** | >500 vs. ≤500 m | **3.66** | **2.02** | **2.35** | **0.019** | **1.24-10.78** |
| **Travel to non-endemic area** | yes vs. no | **0.41** | **0.22** | **-1.66** | **0.097** | **0.15-1.17** |
| **Travel to endemic area** | yes vs. no | 4.97 | 5.05 | 1.58 | 0.115 | 0.68-36.49 |
| **≥10h/week in mixed forest during leisure time** | yes vs. no | 2.81 | 2.16 | 1.35 | 0.178 | 0.63-12.64 |
| **≥10h/week in cottage gardens** | yes vs. no | **0.24** | **0.20** | **-1.75** | **0.079** | **0.05-1.18** |

Backwards selection procedure with p=0.05 as the cut off was used to obtain the final model. Travel to endemic areas was forced into the final model due to the importance of this factor.
