# Supplementary material for: A National Case-Control Study Identifies Human Socio-Economic Status and Activities as Risk Factors for Tick-Borne Encephalitis in Poland
Source: PLoS One. 2012 Sep 19;7(9):e45511. doi: 10.1371/journal.pone.0045511 (PMC3446880; doi:10.1371/journal.pone.0045511)
Supplement: Text S1 — Rationale for figures and tables below. (DOCX) [file pone.0045511.s020.docx]

**Organization of the study and definition of endemic areas, Poland, 2009.**

Table S1 shows the rationale of recruiting study teams in 10 of 16 Polish provinces. See also supplementary Figure S1, a map of the provinces included in the study, with the definition of an endemic area, and Figure S2, a map of endemic areas and the number of cases recruited to the study by administrative district.

**Summary of all variables included in the univariate analysis** for residents of endemic (Table S2) and non-endemic regions (Table S3).

**Selection of variables for the final model**

The document provides a detailed description of the variable selection procedures used in the national case control study of TBE risk factors.

Tables S4-S10 concern residents of endemic regions. Table S4 summarizes the selection criteria for ordinal variables categorization. Tables S5-S9 contain intermediate multivariate models studying selected groups of variables, and Table S10 displays the results of the main effects pre-final model including all candidate variables.

Similarly, for residents of non-endemic areas, Table S11 summarizes selection criteria for ordinal variables categorization; Tables S12-S16 describe results of intermediate multivariate models; and Table S17 shows the results of the main effects pre-final model including all candidate variables.

**Residents of endemic areas**

Based on univariate statistics (Table S2) the following variables were excluded due to limited occurrence (small sample size): having sheep, goats or cows in the farm, immunization status, leisure time spent in deciduous or coniferous forests, time spent in town parks in relation to work, drinking unpasteurized sheep milk, place of exposure to ticks. Collapsing occupation categories was also considered.

**Residents of non-endemic areas**

Based on univariate statistics (Table S3) several variables were excluded from further analysis due to small numbers. The most efficient categorization of ordered variables was selected based on AIC and BIC criteria (Table S11).
